# A screening of inhibitors targeting the receptor kinase FERONIA reveals small molecules that enhance plant root immunity

**DOI:** 10.1111/pbi.13925

**Published:** 2022-10-09

**Authors:** Hong‐Bin Liu, Xiaoxu Li, Jun Cai, Ling‐Li Jiang, Xin Zhang, Dousheng Wu, Lifeng Wang, Aiguo Yang, Cun Guo, Jia Chen, Wenxuan Pu, Feng Yu

**Affiliations:** ^1^ State Key Laboratory of Chemo/Biosensing and Chemometrics College of Biology, Hunan University Changsha China; ^2^ Technology Center China Tobacco Hunan Industrial Co., Ltd. Changsha China; ^3^ State Key Laboratory of Crop Stress Adaptation and Improvement, School of Life Sciences Henan University Kaifeng China; ^4^ State key Laboratory of Hybrid Rice, Hunan Agricultural Biotechnology Research Institute Hunan Academy of Agricultural Sciences Changsha China; ^5^ Key Laboratory for Tobacco Gene Resources, Tobacco Research Institute Chinese Academy of Agricultural Sciences Qingdao China; ^6^ Yuelushan Laboratory Changsha China

**Keywords:** inhibitors, plant resistance, receptor kinase FERONIA, root immunity, soil‐borne diseases

## Abstract

Receptor‐like kinases (RLKs) constitute the largest receptor family involved in the regulation of plant immunity and growth, but small‐molecule inhibitors that target RLKs to improve agronomic traits remain unexplored. The RLK member FERONIA (FER) negatively regulates plant resistance to certain soil‐borne diseases that are difficult to control and cause huge losses in crop yields and economy. Here, we identified 33 highly effective FER kinase inhibitors from 1494 small molecules by monitoring FER autophosphorylation *in vitro*. Four representative inhibitors (reversine, cenisertib, staurosporine and lavendustin A) inhibited the kinase activity of FER and its homologues in several crops by targeting the conserved ATP pocket in the kinase structure. FER contributes to the physiological impact of representative inhibitors in plants. The treatment of roots with reversine, staurosporine and lavendustin A enhanced innate immunity in plant roots and thus alleviated soil‐borne diseases in tobacco, tomato and rice without growth penalties. Consistently, RNA sequencing assays showed that lavendustin A and reversine exert profound impacts on immunity‐related gene expression. Our results will set a new milestone in the development of the plant RLK kinase regulation theory and provide a novel strategy for the prevention and control of plant soil‐borne diseases without growth penalties.

## Introduction

Plants face a trade‐off between immunity and growth, and the establishment of crops with both high resistance and high yield is intrinsically challenging (Wang *et al*., 2021). Small molecules can be used for a set time to avoid prolonged stimulation of plant immunity and thus impact growth, and some small molecules may even promote plant resistance and growth simultaneously (Qiu *et al*., 2017). Their targeting of key agricultural target genes can, in principle, address the dilemma of coaxing high‐yielding plant varieties with low resistance towards more resistant states on demand (Marshall *et al*., 2012; Vaidya *et al*., 2019).

Receptor‐like kinases (RLKs) form the largest receptor family in plants (Franck *et al*., 2018; Yu and Luan, 2016). Approximately 600 and 1000 RLK family members have been found in *Arabidopsis thaliana* and *Oryza sativa*, respectively (Li *et al*., 2016). A large number of studies have shown that RLKs are involved in the regulation of plant resistance and growth and play key roles in signal sensing and transduction (Kanyuka and Rudd, 2019; Zhong and Qu, 2019). A common working mode of a plasma membrane‐localized RLK is as follows: the RLK senses and recognizes ligand molecules, such as microbe‐associated molecular patterns (MAMPs), peptides or hormones, through the extracellular domain and transmits the extracellular signal into the cell. This process triggers phosphorylation of the intracellular kinase domain, recruits specific substrates and initiates a signal cascade amplification effect (Chen *et al*., 2020). Thus, the kinase activity of RLKs is likely to be essential for their function. The number and role of RLKs in plants can be compared with those of G protein‐coupled receptors (GPCRs) in humans (Shiu and Bleecker, 2001). Based on the many successful examples of drug molecules developed for GPCRs (Attwood *et al*., 2021), the use of small molecules (inhibitors or agonists) to manipulate RLK kinase activity and thus regulate downstream signals and control agronomic traits is scientifically reasonable and significant. However, to the best of our knowledge, no previous study has screened RLK inhibitors or agonists or explored their applications.

FERONIA (FER), an important member of the RLK subfamily of *Catharanthus roseus* RLK1‐like (*Cr*RLK1L), is a key regulator of cell growth and stress response (Stegmann *et al*., 2017; Zhu *et al*., 2021). FER negatively regulates plant resistance to certain pathogens, particularly soil‐borne pathogens. For example, FER mutation can significantly inhibit the invasion of plants by the typical soil‐borne pathogens *Meloidogyne incognita* and *Fusarium oxysporum* (Masachis *et al*., 2016; Zhang *et al*., 2020a). Both *M*. *incognita* and *F*. *oxysporum* secrete peptides with functions similar to those of the FER ligand rapid alkalinization factor (RALF), which can inhibit plant immune responses and change the cell pH by hijacking the FER receptor on the plant cell membrane to promote infection (Zhang *et al*., 2020a). The FER mutant exhibits strong resistance to the leaf pathogen powdery mildew (Kessler *et al*., 2010) and deletion of the homologous FER protein FLR2 or FLR11 in rice enhances rice blast resistance without a growth penalty (Yang *et al*., 2020). Moreover, FER function is likely evolutionarily conserved across plant species (Zhang *et al*., 2020b). For example, FLR1 mutation can improve the resistance of rice to root‐knot nematodes (Zhang *et al*., 2020a). GmLMM1, which shares high similarity in terms of sequence and function with FER, forms an immune complex with FLS2‐BAK1 to regulate pattern‐triggered immunity in soybean (*Glycine max*, Wang, Liang *et al*., 2020) and GmLMM1 mutants exhibit enhanced resistance against *M. incognita* (Zhang *et al*., 2021). Therefore, FER is a potential target in the prevention of plant diseases, particularly soil‐borne diseases.

There is a large difference between root immunity and leaf immunity and root immunity is less understood, which may be due to the extremely complex soil environment (Dodds and Rathjen, 2010). Therefore, soil‐borne diseases are very difficult to control, and green and efficient prevention strategies are lacking. These diseases can cause reductions in crop yields of 20%–40% and, in severe cases, 60%–100% and thus are a bottleneck in sustainable agricultural development (Bakker *et al*., 2020). In this study, given that FER negatively regulates plant resistance to certain soil‐borne diseases, we conducted high‐throughput screening of small‐molecule inhibitors targeting FER. We found that several FER inhibitors enhanced the defence response in plant roots to improve resistance to major soil‐borne diseases.

## Results

### High‐throughput screening of FER kinase inhibitors

**Figure 1:**
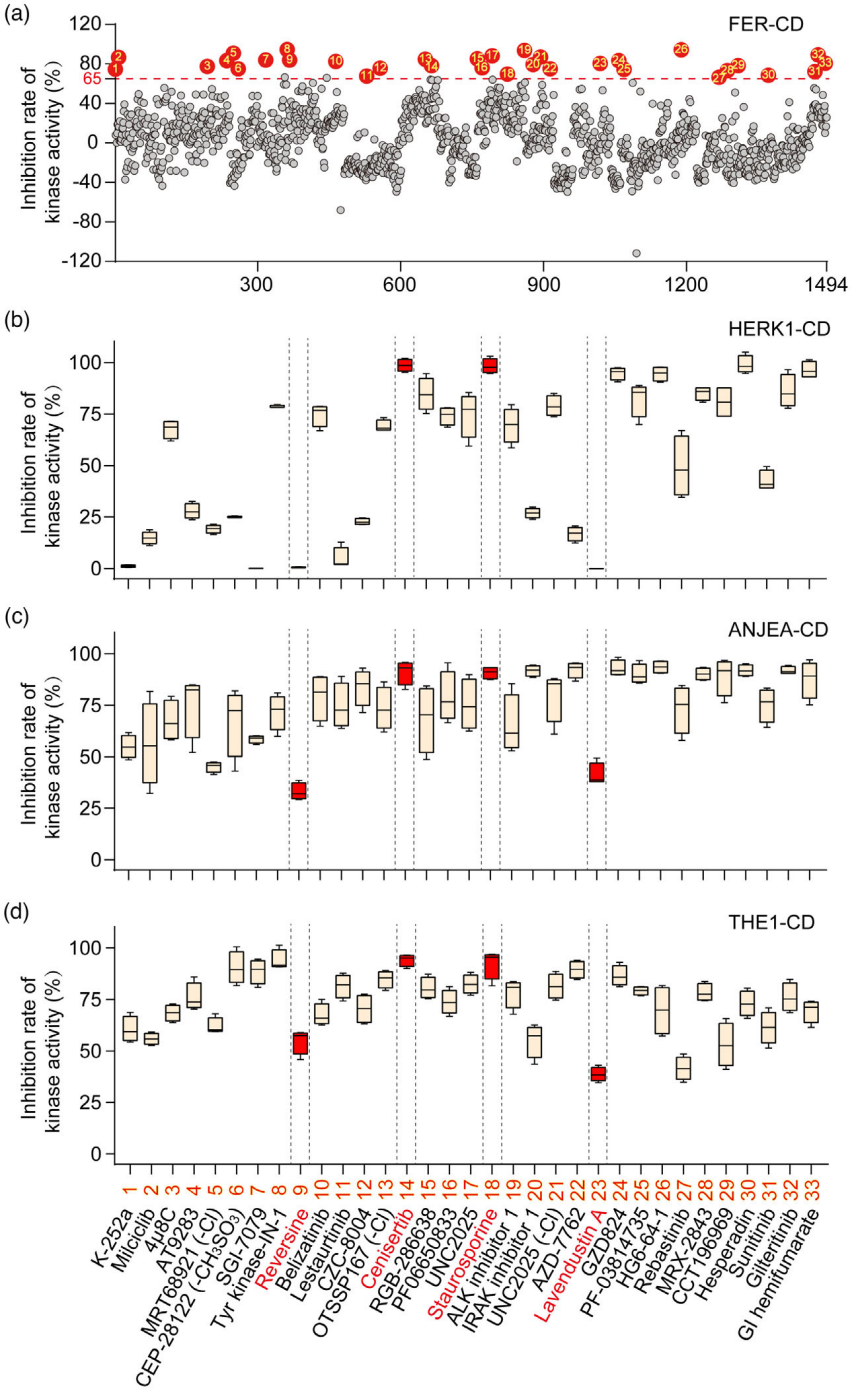
High‐throughput screening of FERONIA kinase inhibitors. (a) Effect of 1494 small molecules (5 μm) on FER cytoplasmic domain (FER‐CD; 0.5 μm) kinase activity (autophosphorylation). The consumption of ATP (μm) in 10 min was used to indicate the level of kinase activity, and DMSO (0.05%) was used as a negative control. An inhibition rate of 65% was used as the criterion for screening FER inhibitors. The data are presented as the means (*n* = 4). Three independent experiments were performed for small molecules with inhibition rates >65%, and similar results were obtained. (b–d) Effects of 33 FER inhibitors on the kinase activity of the FER family proteins HERK1‐CD (b), ANJEA‐CD (c) and THE1‐CD (d). The FER inhibitors with relative specificity for FER and high efficiency in inhibiting the activity of FER family proteins are marked in red. DMSO (0.05%; v/v) was used as a negative control. In boxplots, the middle line represents the median, box edges delimit lower and upper quartiles, and whiskers show the highest and lowest data points (*n* = 4). The assay was repeated three times with similar results.

We conducted a high‐throughput screening of 1494 potential kinase inhibitors to identify small molecules that inhibit FER kinase activity/autophosphorylation. After FER cytoplasmic domain (FER‐CD) kinase activity was measured to equal 1.10 μm (Figure S1a,b), we established an *in vitro* high‐throughput screening system (Figure S1c) and identified 33 FER kinase inhibitors with ≥65% inhibition (Figure 1a; Figure S2). To preliminarily assess the specificity of FER inhibitors, the kinase activities of three FER family proteins HERK1‐CD, ANJEA‐CD and THE1‐CD were measured to equal 2.48, 1.12 and 1.55 μm (2.25‐, 1.02‐ and 1.41‐fold higher than FER‐CD activity), respectively (Figure S1a,b). Further testing revealed that reversine and lavendustin A exhibited relative specificity for FER‐CD and that their inhibition rates for the three family proteins were all lower than 55%, whereas cenisertib and staurosporine also inhibited the kinase activities of these family proteins very efficiently, with all the inhibition rates higher than 88% (Figure 1b–d). These four FER inhibitors were used as representatives in subsequent studies. Their structure and application information are summarized in Table 1. It is worth mentioning that staurosporine and lavendustin A were isolated from *Streptomyces staurosporeus* and *S*. *griseolavendus* in soil, respectively (Onoda *et al*., 1989; Yoshizawa *et al*., 1990). To determine whether FER inhibitors work in plants, we performed *in vivo* FER phosphorylation assay using FER‐specific anti‐phosphorylation antibodies. Since the background phosphorylation level of FER in Arabidopsis is difficult to detect, we used RALF1 to activate the phosphorylation of FER. The results showed that treatments of the four representative FER inhibitors at a concentration of 5 μm reduced RALF1‐induced FER phosphorylation compared with the negative controls dimethyl sulfoxide (DMSO) and inactive small molecule lapatinib which did not affect FER kinase activity (Figure S3a). This indicates the potential application of FER inhibitors in plants.

**Table 1:** Structure and application information of the four FER inhibitors investigated in this study

Name	CAS Number	Chemical structure	Application
Reversine	656820‐32‐5	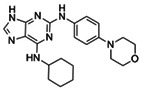 C_21_H_27_N_7_O	Reversine is a novel class of ATP‐competitive Aurora kinase inhibitor with IC50s of 400, 500 and 400 nm for Aurora A, Aurora B and Aurora C, respectively (D'Alise *et al*., 2008)
Cenisertib	871357‐89‐0	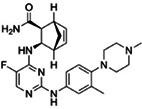 C_24_H_30_FN_7_O	Cenisertib (AS‐703569) is a multi‐kinase inhibitor that blocks the activity of Aurora‐kinase‐A/B, ABL1, AKT, STAT5 and FLT3 (McLaughlin *et al*., 2010)
Staurosporine	62996‐74‐1	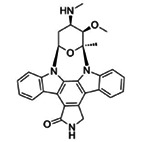 C_28_H_26_N_4_O_3_	Staurosporine is a potent and non‐selective inhibitor of protein kinases with IC50s of 6 nm, 15 nm, 2 nm, and 3 nm for PKC, PKA, c‐Fgr, and Phosphorylase kinase respectively (Omura *et al*., 2018)
Lavendustin A	125697‐92‐9	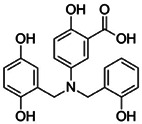 C_21_H_19_NO_6_	Lavendustin A (RG‐14355), isolated from *Streptomyces Griseolavendus*, is a potent, specific and ATP‐competitive inhibitor of tyrosine kinase, with an IC50 of 11 ng/mL for EGFR‐associated tyrosine kinase (Hsu *et al*., 1991)

### FER inhibitors target the ATP pockets of FER and its homologues in crops

**Figure 2:**
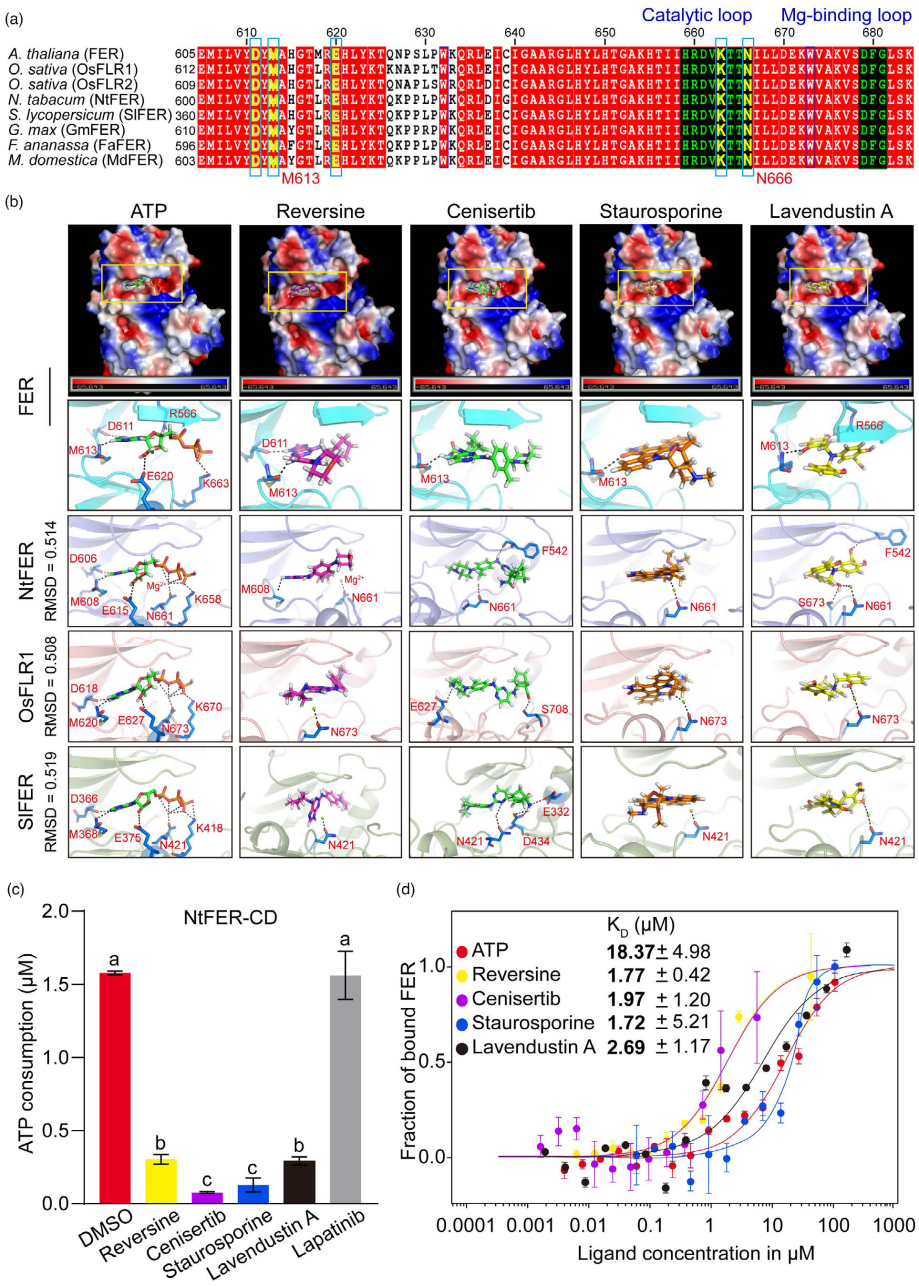
FER inhibitors target the ATP pocket of FER and its homologues in crops. (a) Multiple sequence alignment of partial kinase domains of FER and its homologues in the crops *Oryza sativa* (OsFLR1 and OsFLR2), *Nicotiana tabacum* (NtFER), *Solanum lycopersicum* (SlFER), *Fragaria* × *ananassa* (FaFER), *Glycine max* (GmFER) and *Malus domestica* (MdFER). (b) Molecular docking between FER inhibitors (reversine, cenisertib, staurosporine and lavendustin A) and FER and its homologues (NtFER, OsFLR1 and SlFER). The kinase structures of FER homologues were obtained by homology modelling, and the root mean squared deviation (RMSD) indicates the similarity of the spatial structure of NtFER, FLR1 and SlFER to that of FER. Semiflexible docking was performed, and ATP was used as a positive control. The inactive small molecule lapatinib could not dock successfully. The black dashed lines indicate polar interactions, and the targets of ATP or inhibitors on FER are marked in red. (c) Inhibitory effects of four FER inhibitors on the kinase activity of the homologous FER protein NtFER‐CD in tobacco. DMSO and lapatinib were used as negative controls. The data are shown as the means ± SDs (*n* = 4) and different letters above the bars indicate significant differences (*P* < 0.05) determined by ANOVA with Tukey's HSD test. The assay was repeated three times with similar results. (d) Quantification of the binding affinity between fluorescently labelled FER‐CD and FER inhibitors using MST. ATP and the inactive small‐molecule lapatinib were used as positive and negative contrls, respectively. The data points indicate the fraction of inhibitor‐ or ATP‐bound FER (▵Normal/Amplitude) at different ligand concentrations, and the curves indicate the calculated fits. The affinity curve could not be fitted with lapatinib. The data are shown as the means ± SEs (*n* = 3) and the mean values of the equilibrium dissociation constants (K_D_) are shown in the panel. Three independent experiments were performed with similar results.

We performed a multiple sequence alignment of the CDs (including the juxtamembrane region, kinase region and carboxy‐terminal region) of Arabidopsis FER and FER homologues in various crops. The results showed that the kinase domain sequences had a high degree of similarity (Figure S3b; Figure 2a). The sequences of key functional structures, namely, the β3 lysine, catalytic loop and Mg‐binding loop, were exactly the same, and only one or two amino acids were different in the C helix and activation loop sequences (Figure S3b; Figure 2a). These results indicate that the FER kinase domain is relatively conserved in crops.

The FER inhibitors reversine, cenisertib, staurosporine and lavendustin A work as ATP competitors in animal systems (Long *et al*., 2021). To test whether these inhibitors also inhibit FER activity in a similar manner, we analysed the activity positions of the small molecules in FER and its homologues in tobacco (*Nicotiana tabacum*; NtFER), rice (OsFLR1), and tomato (*Solanum lycopersicum*; SlFER) via molecular docking. The results showed that all four inhibitors were well connected to the ATP‐binding pocket of FER or its homologues (Figure 2b), and the control lapatinib, which cannot inhibit FER, could not dock successfully. The root mean square deviation (RMSD) values of the three‐dimensional structure of NtFER, OsFLR1 and SlFER compared with the three‐dimensional structure of FER were 0.514, 0.508 and 0.519, respectively (Figure 2b), which indicated that the spatial structure of NtFER, OsFLR1 and SlFER is highly similar to that of FER. We also found that the sites where NtFER, OsFLR1 and SlFER interact with ATP may be conserved aspartic acid (D) 611, methionine (M) 613, glutamic acid (E) 620, lysine (K) 663 and asparagine (N) 666, and only N666 is replaced by arginine (R) 566 in the site where FER interacts with ATP (the sequence number indicates the corresponding position of the FER sequence; Figure 2b). This conservation was further supported by the finding that these four FER inhibitors can also inhibit the kinase activity of NtFER‐CD with high efficiency and lapatinib was ineffective against NtFER‐CD (Figure 2c). Taken together, these data indicate that FER inhibitors can inhibit FER and its homologues in multiple crop species.

Next, we measured the binding affinity of the four inhibitors to FER via MST. The results showed that the equilibrium dissociation constants (K_D_) of the binding of reversine, cenisertib, staurosporine and lavendustin A to FER were 1.77, 1.97, 1.72 and 2.69 μm, respectively, whereas the K_D_ of the binding of ATP to FER was 18.37 μm (Figure 2d), and the affinity curve could not be fitted with inactive lapatinib. Moreover, the concentration dependence test results revealed that the inhibitory capacity of these inhibitors was concentration‐dependent (Figure S3c). These findings indicate that the four FER inhibitors had a higher affinity for FER than ATP and that they may target the ATP‐binding pocket of FER in a concentration‐dependent manner that competes with ATP.

### Roles of FER inhibitors in modulating soil‐borne diseases and plant growth

**Figure 3:**
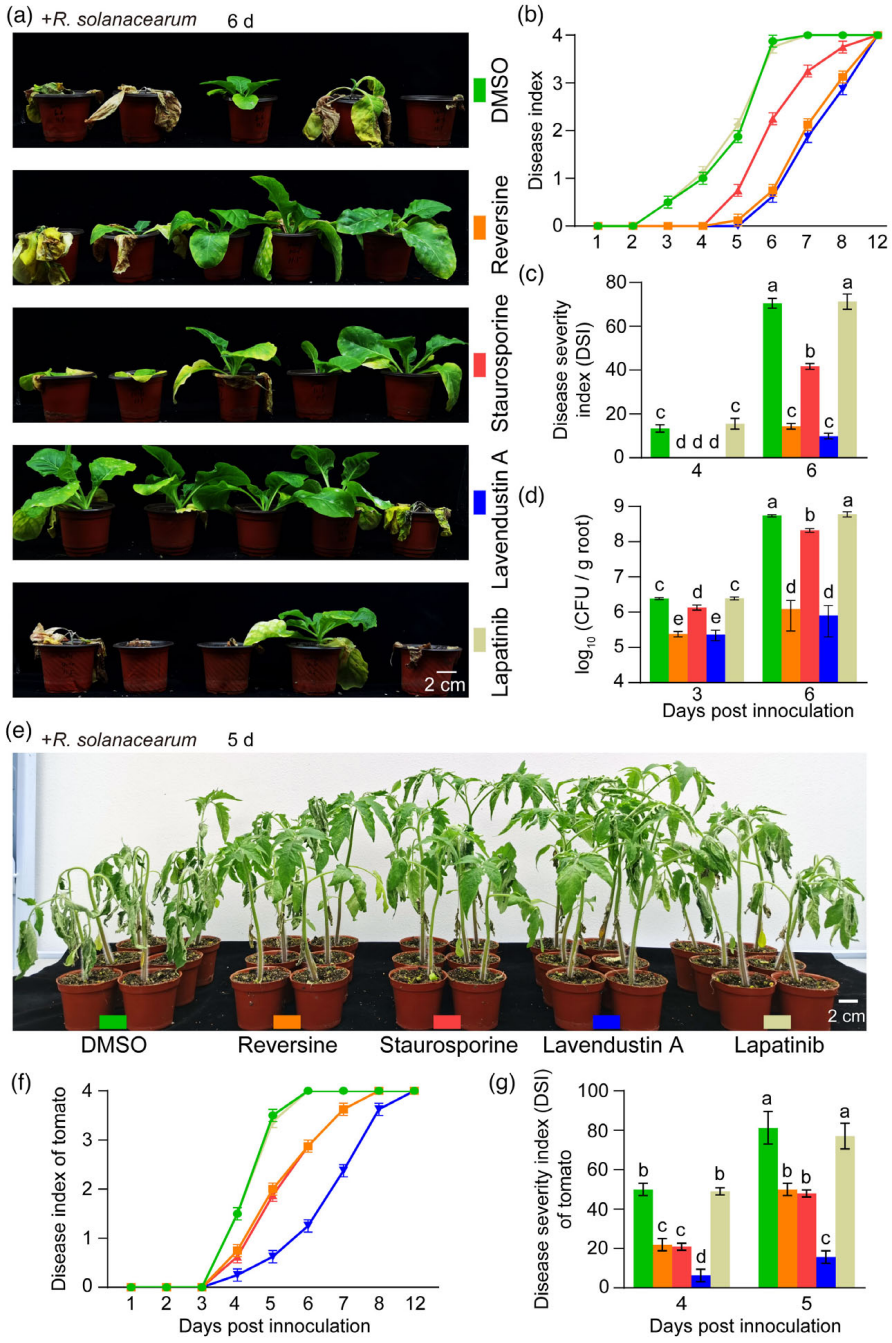
FER inhibitors effectively control tobacco and tomato bacterial wilt. (a) Representative images of four‐week‐old tobacco plants pretreated with 5 μm FER inhibitors 6 days after infection with *Ralstonia solanacearum*. (b and c) Disease index (b) and disease severity index (DSI) (c) of tobacco on the indicated days after inoculation with *R. solanacearum*. (d) Bacterial counts of *R. solanacearum* in the roots of tobacco on the indicated days after inoculation with *R. solanacearum*. (e) Representative images of 4‐week‐old tomato plants pretreated with 5 μm FER inhibitors 5 days after *R*. *solanacearum* infection. (f and g) Disease index (f) and disease severity index (DSI) (g) of tomato on the indicated days after inoculation with *R. solanacearum*. The assays were performed in triplicate using 30 individual tobacco plants or 18 individual tomato plants per treatment. DMSO (0.05%; v/v) and lapatinib were used as negative controls. In (b–d, f and g), the data shown indicate the means ± SDs of three biological replicates. In (c, d and g), different letters above the bars indicate significant differences (*P* < 0.05) determined by ANOVA with Tukey's HSD test.

**Figure 4:**
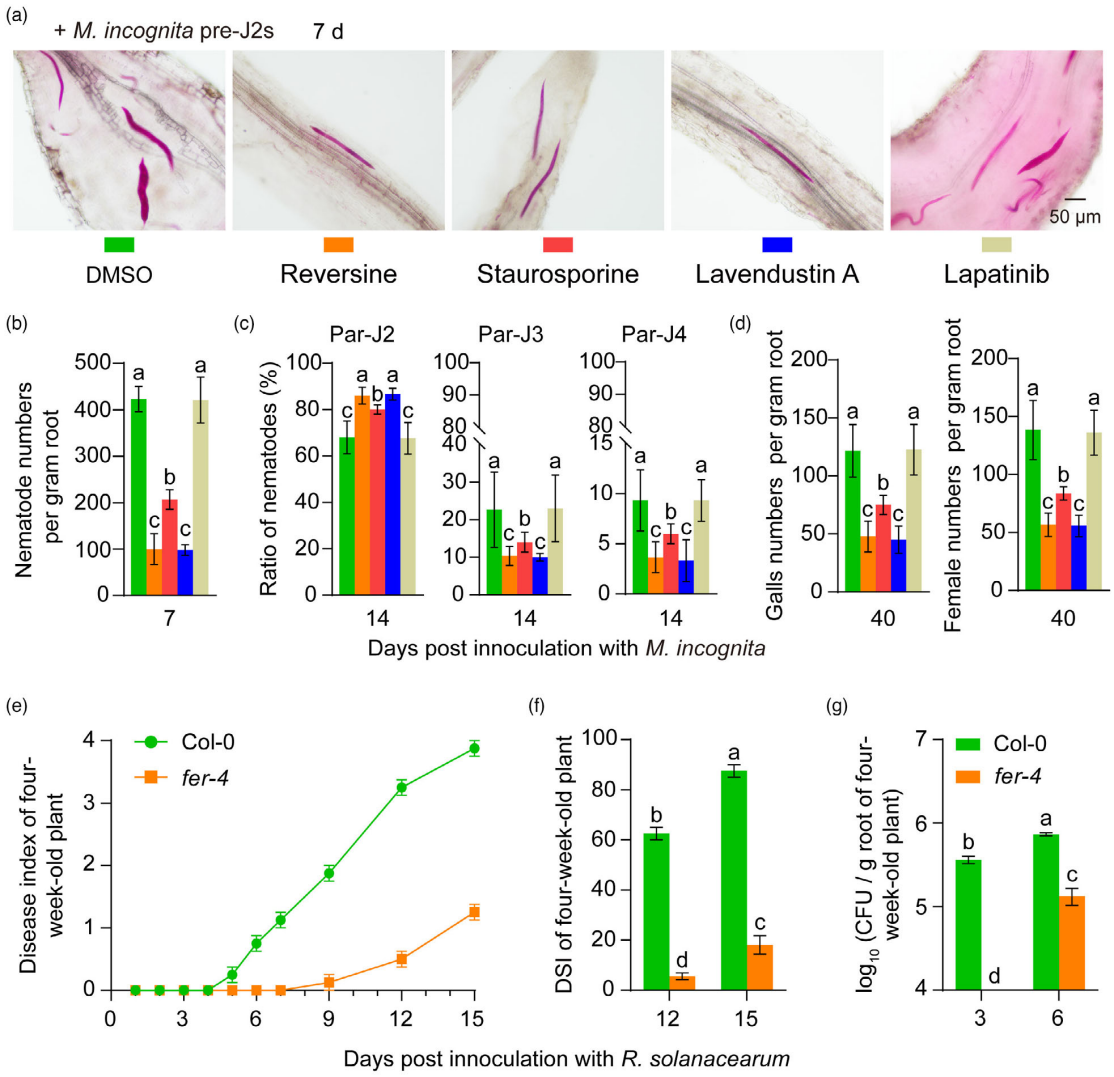
FER inhibitors alleviate rice root‐knot nematode disease and FER mutations enhance Arabidopsis resistance to bacterial wilt. (a) Representative images of fuchsin‐stained *Meloidogyne incognita* in the roots of rice plants pretreated with 5 μm FER inhibitors at 7 days post‐inoculation with 800 *M*. *incognita* pre‐J2s. (b) Number of nematodes per gram of root at 7 days after inoculation. (c) The ratio of par‐J2, par‐J3 and par‐J4 nematodes to the total number of parasitic nematodes at 14 days after inoculation. (d) Gall and female numbers per gram of plant roots at 40 dpi. The assay was performed in triplicate using 18 individual rice plants per treatment. DMSO (0.05%; v/v) and lapatinib were used as negative controls. (e and f) Disease index (e) and disease severity index (DSI) (f) of four‐week‐old Arabidopsis Col‐0 and *fer‐4* on the indicated days after inoculation with *R. solanacearum*. (g) Bacterial counts of *R. solanacearum* in the roots of Col‐0 and *fer‐4* on the indicated days after inoculation with bacteria at 3 × 10^8^ CFU/mL. The assay was performed in triplicate using 24 individual Arabidopsis plants of each genotype. In (b–g), the data shown indicate the means ± SDs of three biological replicates. In (b–d, f and g), different letters above the bars indicate significant differences (*P* < 0.05) determined by ANOVA with Tukey's HSD test.

FER inhibitors are speculated to prevent soil‐borne diseases. Bacterial wilt and root‐knot nematode disease are serious soil‐borne diseases. Thus, we selected two representative soil‐borne pathogens, *Ralstonia solanacearum* and *M*. *incognita*, to test our hypothesis. We first detected the changes in the resistance of Arabidopsis seedlings to *R*. *solanacearum* after pretreatment with 5 μm FER inhibitors. The results showed that reversine, staurosporine and lavendustin A effectively prevented the cotyledon bleaching caused by *R. solanacearum*, whereas the cotyledons of seedlings treated with cenisertib and the negative controls DMSO and lapatinib turned white 4 days after inoculation with the bacteria (Figure S4a). We then tested the control effects of reversine, staurosporine and lavendustin A against typical crop soil‐borne diseases: tobacco and tomato bacterial wilt and rice root‐knot nematode disease. The results showed that all three inhibitor treatments, particularly the reversine and lavendustin A treatments, alleviated tobacco bacterial wilt (Figure 3a–d). The three FER inhibitors also reduced the susceptibility of tomato to *R. solanacearum*, with lavendustin A exhibiting the best control effect (Figure 3e–g). We also found that these inhibitors slowed the development of rice root‐knot nematode disease and reversine and lavendustin A showed better preventive effects than staurosporine (Figure 4a–d).

Previous studies have shown that FER mutants exhibit resistance to *M*. *incognita* in Arabidopsis, rice and soybean (Zhang *et al*., 2020a, 2021). We assessed the response of FER mutants to *R. solanacearum*, which has not been reported until now. The results showed that the root elongation inhibition rates in *fer‐4* (complete loss of FER function) and *fer‐5* (partial loss of FER function) seedlings were significantly lower than that in the wild‐type Columbia (Col‐0; Figure S4b,c) and that four‐week‐old *fer‐4* plants grown in soils also exhibited stronger resistance to *R. solanacearum* than Col‐0 (Figure S4d; Figure 4e–g). These results indicate that FER also negatively regulates plant resistance to *R. solanacearum*.

**Figure 5:**
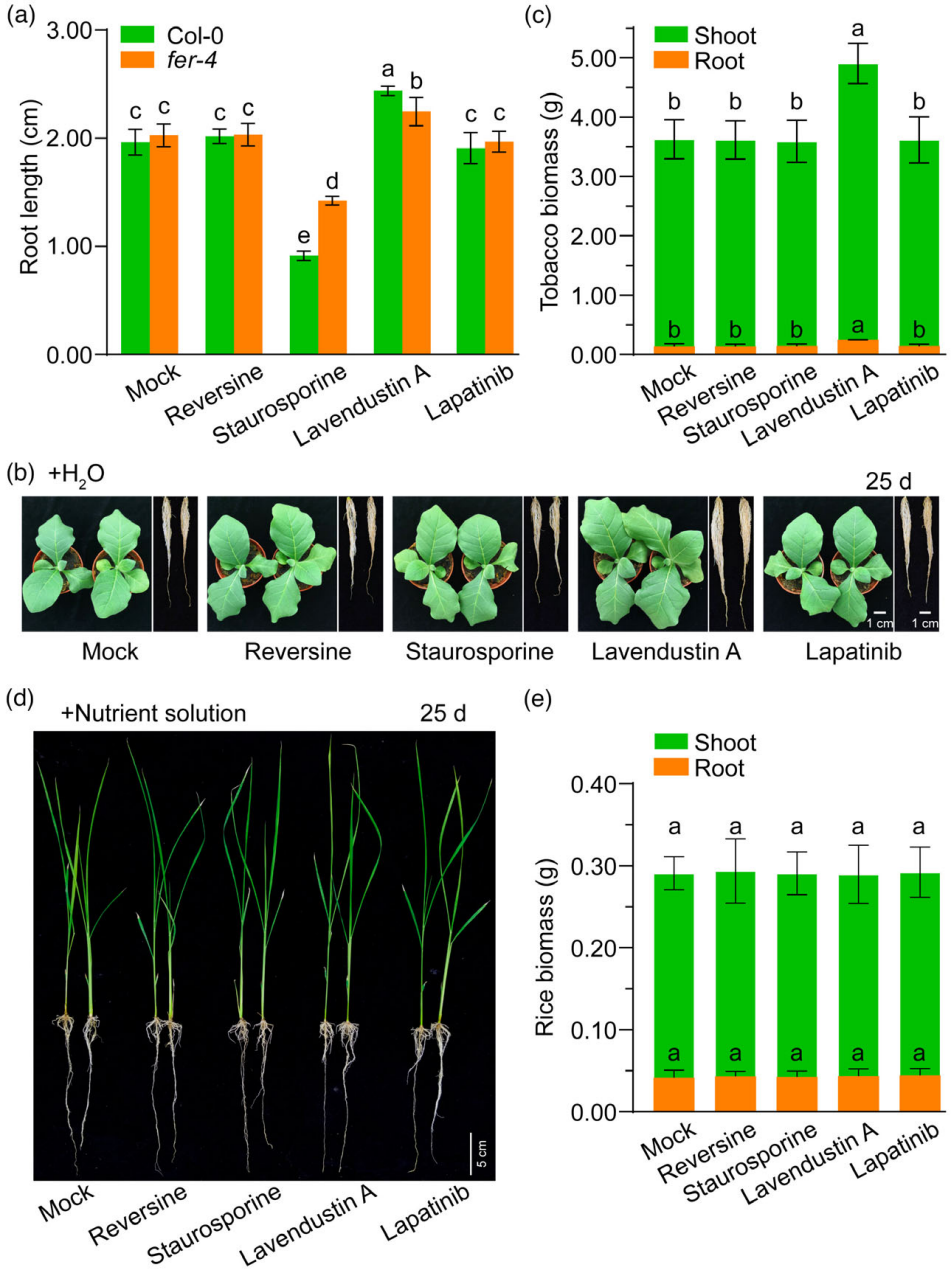
Impact of FER inhibitors on plant growth. (a) Quantification of root length of *Arabidopsis thaliana* Col‐0 and *fer‐4* seedlings treated with 5 μm FER inhibitors for 3 days. (b and d) Representative images of shoots and roots of tobacco (b) and rice (d) on Day 25 after treatment with FER inhibitors. (c and e) Shoot and root biomass (fresh weight) of tobacco (c) and rice (e) on Day 25. ddH_2_O (mock) and the inactive small‐molecule lapatinib were used as negative controls. The data are presented as the means ± SDs (*n* = 12 for Col‐0 and *fer‐4*; *n* = 6 for tobacco and rice), and three independent experiments yielded similar results. Different letters above the bars indicate significant differences (*P* < 0.05) determined by ANOVA with Tukey's HSD test.

We further investigated whether FER inhibitor application exerts adverse effects on plant growth. The results showed that compared with the effects obtained with the controls ddH_2_O (mock) and lapatinib, reversine did not affect the growth of roots and shoots of Arabidopsis Col‐0 seedlings, staurosporine severely inhibited root and shoot growth, and lavendustin A significantly promoted root and shoot growth (Figure S5a; Figure 5a). Simultaneous determination of the growth of *fer‐4* roots and shoots revealed that the inhibitory and promotive effects of staurosporine and lavendustin A, respectively, were partly dependent on FER (Figure 5a; Figure S5a,b). For crop growth, we found that lavendustin A significantly improved the growth of tobacco shoots and roots, whereas reversine and staurosporine did not observably affect the biomass of tobacco shoots or roots compared with the controls (Figure 5b,c). Rice biomass data revealed that none of the three FER inhibitors caused changes in the growth of rice shoots and roots relative to the controls (Figure 5d,e). These results suggest that FER inhibitors did not restrict the normal growth of the crop species tested in our work.

### FER is involved in the FER inhibitor action in enhancing plant root immunity

**Figure 6:**
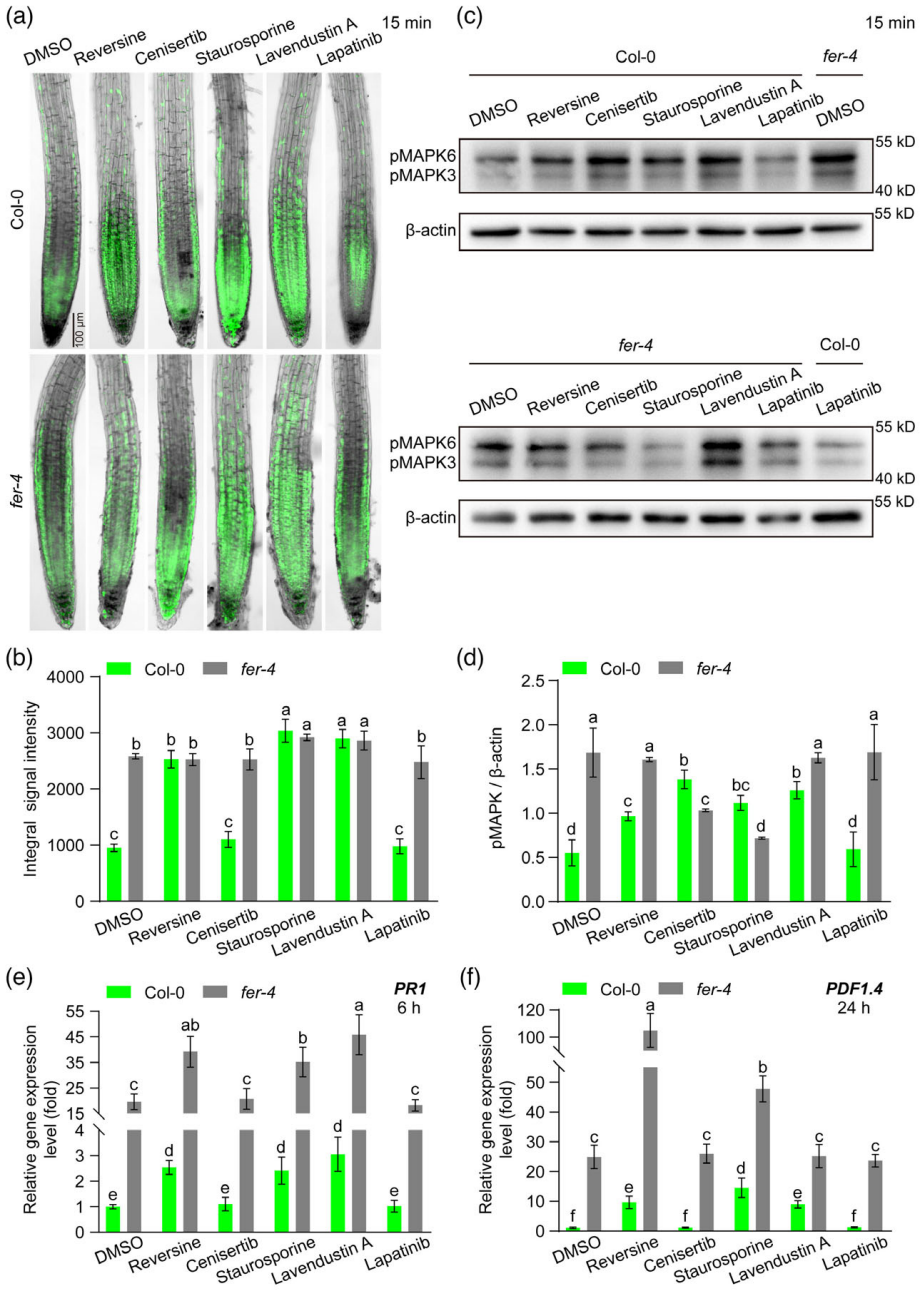
FER is involved in the roles of FER inhibitors in enhancing plant root immunity. (a) Representative images of H_2_DCF‐DA‐stained roots of Col‐0 and *fer‐4* treated with 5 μm FER inhibitors for 15 min. (b) Quantified integral H_2_DCF‐DA signal intensity in the roots. (c) Western blot analysis of MAPK phosphorylation in Col‐0 and *fer‐4* seedling roots treated with 5 μm FER inhibitors for 15 min. (d) Relative quantification of MAPK phosphorylation levels (pMAPK/β‐actin). (e and f) Relative expression levels of *PR1* (e) and *PDF1.4* (f) in Col‐0 and *fer‐4* seedling roots treated with 5 μm FER inhibitors for 6 or 24 h. DMSO (0.05%; v/v) and the inactive small‐molecule lapatinib were used as negative controls. In (b and d–f), the data are presented as the means ± SDs of three biological replicates and different letters above the bars indicate significant differences (*P* < 0.05) determined by ANOVA with Tukey's HSD test.

To understand the mechanisms through which FER inhibitors promote plant resistance to soil‐borne diseases, we analysed the changes in plant root immune capacity after ruling out the possibility of the inhibitors themselves being toxic to pathogens (Figure S5c,d). The reactive oxygen species (ROS) burst, the amplification of phosphorylated mitogen‐activated protein kinase (MAPK) cascade signals, and the transcriptional activation of defence‐related genes [such as salicylic acid (SA)‐dependent *PR1* (*PATHOGENESIS‐RELATED GENE 1*), jasmonic acid (JA)‐mediated *PDF1.4* (*PLANT DEFENSIN 1.4*), MAMP‐induced *FRK1* (*FLG22‐INDUCED RECEPTOR‐LIKE KINASE 1*) and *PER5* (*PEROXIDASE 5*)] are the main innate immune responses in plants (Yuan *et al*., 2021; Zhou *et al*., 2020). Our results showed that reversine and, in particular, staurosporine and lavendustin A significantly increased the production of ROS in Col‐0 roots, whereas cenisertib had no effect on ROS levels compared with the controls DMSO and lapatinib (Figure 6a,b; Figure S6a). We also found a stronger ROS burst in *fer‐4* or *srn* roots than in Col‐0 or C24 roots (Figure S6c), and the enhancement in the ROS level obtained with the three FER inhibitors was partly dependent on FER (Figure 6b; Figure S6b). We further found that the increased MAPK phosphorylation and expression levels of *PR1* and *PDF1.4* in the roots induced by these inhibitors depended or partially depended on FER (Figure 6c–f; Figure S6d; Figure S7a–d). Confocal microscopy data indicated that staurosporine observably enhanced the expression of *FRK1*, elevated the expression of *PER5* to a certain extent and caused expansion of the root cells. Cenisertib increased the expression of *FRK1* but did not affect the expression of *PER5*. Reversine and lavendustin A, similar to the negative controls DMSO and lapatinib, did not affect the expression levels of these two genes in the roots under our processing conditions (Figure S7e,f). These results indicate that certain FER inhibitors enhance plant root immunity through a mechanism, that is partially dependent on FER.

### Lavendustin A and reversine promote the expression of defence‐related genes in tobacco roots

**Figure 7:**
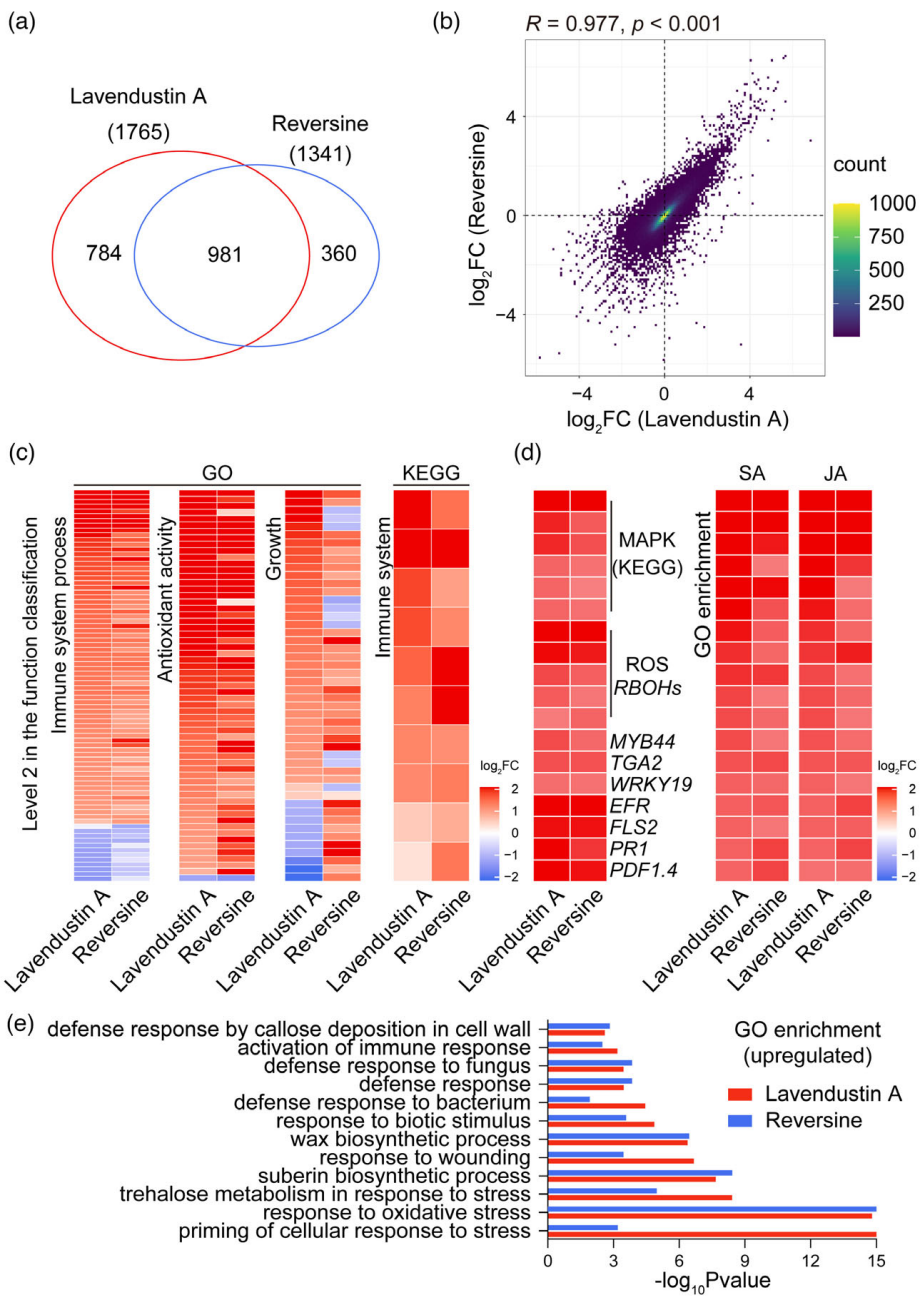
Lavendustin A and reversine promote the expression of defence‐related genes in tobacco roots. (a) Venn diagrams of significantly differentially expressed genes (DEGs) under lavendustin A and reversine treatments relative to the DMSO‐treated control. The criteria of *P* < 0.05 and a fold change >2 or <0.5 were used to identify significant DEGs (*n* = 4). (b) Spearman's correlation analysis of DEGs in tobacco roots treated with lavendustin A and reversine compared with those treated with the control DMSO (*n* = 4). The *x*‐ and *y*‐axes indicate changes in the inclusion level; the yellow‐green area indicates a high density. (c) Fold change of DEGs in different GO and KEGG classifications (level 2). (d) Fold change of DEGs in the MAPK signalling pathway enriched by KEGG or the main DEGs related to ROS synthesis and immunity and DEGs related to the defence hormones salicylic acid (SA) and jasmonic acid (JA) enriched by GO. (e) GO enrichment analysis based on the *P* values for genes that showed similar up‐regulation with lavendustin A and reversine treatments compared with the DMSO treatment.

To further explore the molecular mechanism of FER inhibitors in inducing disease resistance in crop roots without negatively affecting plant growth, we analysed the transcriptome changes in four‐week‐old tobacco roots treated with the highly effective inhibitors lavendustin A and reversine via RNA‐seq. We observed strong changes in the root gene expression profile after treatment with lavendustin A or reversine (Figure S8a,b). We found a large overlap of 981 significantly differentially expressed genes (DEGs) between these two comparison groups (Figure 7a). Interestingly, most of the transcript changes caused by lavendustin A and reversine were in the same direction, and the values were highly consistent (*R* = 0.977, *P* < 0.001; Figure 7b). Furthermore, a clustering analysis showed that the expression changes in most DEGs detected in tobacco roots treated with lavendustin A and reversine were correlated and changed in the same direction (Figure S8c).

Based on the Gene Ontology (GO) classification of functions, we identified genes that were coregulated by lavendustin A and reversine and were involved in the two functions (immune system process and antioxidant activity) most directly related to disease resistance. Our results showed that 70 of 82 immune genes and 60 of 61 antioxidant genes were up‐regulated by lavendustin A or reversine (Figure 7c; Table S1). Importantly, we found that lavendustin A and reversine differentially elevated the expression levels of most growth‐related genes (Figure 7c; Table S1), which might provide clues to explain why these two inhibitors did not restrict crop growth or why lavendustin A promoted growth. According to the Kyoto Encyclopedia of Genes and Genomes (KEGG) classification, we found that the expression levels of all 10 immune system‐related genes that were coregulated by lavendustin A and reversine were increased, and 22 of 29 environmental adaptation‐related genes closely related to the defence response were also up‐regulated by lavendustin A and reversine (Figure 7c; Figure S8d). Moreover, GO and KEGG enrichment analyses showed that both lavendustin A and reversine enhanced the immune defence response of roots to biotic and abiotic stresses, such as amplification of the core signal MAPK that regulates a series of stress resistance pathways; up‐regulation of the four defence hormone responses (SA, JA, abscisic acid and ethylene); production of cutin, suberin and wax as physical barriers; and synthesis of secondary metabolites that antagonize pathogens, such as alkaloids (Figure 7d,e; Figure S8d; Figure S9; Table S2). In addition, lavendustin A and reversine significantly increased the expression levels of ROS synthesis genes (*RBOHs*), immune‐related transcription factor genes (*MYB44*, *TGA2* and *WRKY19*), pattern‐recognition receptor genes (*EFR* and *FLS2*) and resistance genes (*PR1* and *PDF1.4*) in tobacco roots (Figure 7d; Ngou *et al*., 2021). These data suggest that the FER inhibitors lavendustin A and reversine may strengthen the immune response in tobacco roots by promoting the expression of defence‐related genes.

## Discussion

To the best of our knowledge, this manuscript describes the first systematic screening of RLK inhibitors, and only a few studies have used the broad‐spectrum inhibitor K‐252a to explore the function of RLK in plants (Chinchilla *et al*., 2007; Ichimura *et al*., 2000). Compared with classic genetic approaches, small‐molecule screens offer notable advantages in dissecting plant biological processes, such as technical simplicity, low start‐up costs, and most importantly, bypassing the problems of lethality and redundancy (Xuan *et al*., 2013). Our high‐throughput screening of FER inhibitors may constitute a powerful tool for manipulating kinase activity to facilitate more related studies and provide new chemical tools for the prevention and control of plant soil‐borne diseases without negative effects on growth.

Small molecules usually exert off‐target effects (Cui, 2014). Cenisertib is likely to target other important disease‐resistant kinases based on its high inhibition of the kinase activity of the three FER family proteins, which may have resulted in its loss of function in reducing the susceptibility of Arabidopsis seedlings to *R. solanacearum*. Our seemingly contradictory root growth data (the inhibitory and promotive effects of staurosporine and lavendustin A, respectively, on root growth) may also have been caused by different off‐target effects of the inhibitors. ATP‐competitive inhibitors are generally not very specific. However, differential residues in the kinase ATP‐binding site can confer high specificity for ATP‐competitive inhibitors (Cohen *et al*., 2005). Interestingly, we noticed that the relatively specific inhibitors reversine and lavendustin A also exerted a better control effect on tobacco bacterial wilt and rice root‐knot nematode disease, although reversine and the nonspecific inhibitor staurosporine showed similar control effects on tomato bacterial wilt. The molecular docking results showed that lavendustin A had more intermolecular interactions with FER homologues than reversine and staurosporine and that reversine could target the key amino acid M613 in the ATP‐binding pocket of NtFER in tobacco but not SlFER in tomato. Although the binding pockets of the two FER homologues were similar, the different intermolecular interactions caused by their small spatial differences may determine the preference of reversine for certain species. Although we found that reversine and lavendustin A did not affect the kinase activity of immune‐related PEPR1 (PLANT ELICITOR PEPTIDE 1 RECEPTOR 1) and CERK1 (CHITIN ELICITOR RECEPTOR KINASE 1; Figure S10a,b), their inhibitory effect on the activity of more RLKs and/or other types of kinases requires further investigation to determine their specificity. If the identified inhibitors also suppress certain RLKs or kinases that positively regulate plant resistance to certain soil‐borne diseases, the use of inhibitors may be ineffective or even accelerate the development of certain soil‐borne diseases. Therefore, it is important to further study the role of these inhibitors in more soil‐borne diseases. The structures of FER‐inhibitor complexes and more molecular target RLKs urgently need to be resolved, and our small molecules could be used as lead compounds in future studies to optimize their structures according to the binding position with the aim of improving their specificity and effect. In addition, the development of a relatively broad‐spectrum inhibitor belonging to a class of RLK subfamilies would bypass the difficulties of multigene editing to achieve a rapid and convenient functional analysis of some redundant RLKs or subfamily members.

Very importantly, the kinase domains of FER homologues are highly conserved in many crop species, and four representative FER inhibitors target the ATP pocket of FER and its homologues, which means that our FER inhibitors likely exhibit multispecies generality. This conclusion is supported by data showing that FER inhibitors effectively control bacterial wilt in Arabidopsis, tobacco and tomato and root‐knot nematode disease in rice. Although we did not investigate the effects of these inhibitors on leaf diseases, they may also prevent certain leaf diseases, given that they also promoted the expression of *PDF1.4* in Arabidopsis leaves (Figure S10c) and that mutations in FER or its homologues enhance resistance to powdery mildew (Kessler *et al*., 2010) and rice blast (Yang *et al*., 2020). Unfortunately, the three FER inhibitors reversine, staurosporine and lavendustin A could not completely prevent the occurrence of the diseases or cure already diseased plants under our experimental conditions (Figure S11). Although we illuminated that the inhibitors increase plant immunity via the ROS burst, MAPK signal amplification and up‐regulation of defence‐related genes, partly through FER, they may not exert activity for a long time due to plant metabolism under our processing conditions according to the normal expression level of *PDF1.4* in Arabidopsis roots treated for 96 h (except for staurosporine treatment; Figure S10d). Increased use of FER inhibitors is likely to strengthen disease control, and root treatment may also contribute to systemic acquired resistance given that these inhibitors significantly promoted the expression of SA‐dependent *PR1* and JA‐mediated *PDF1.4*. When root‐knot nematodes secrete peptide RALF‐likes to suppress host immunity via FER (Zhang *et al*., 2020a), FER inhibitors are likely to block this susceptibility signalling pathway to improve plant resistance.

The FER inhibitors staurosporine and lavendustin A were isolated from *S*. *staurosporeus* and *S*. *griseolavendus* in the soil, respectively (Onoda *et al*., 1989; Yoshizawa *et al*., 1990), which led us to speculate that FER may mediate the connection between plant roots and *Streptomyces*, given that FER is involved in interactions between plants and a variety of microorganisms (Masachis *et al*., 2016; Song *et al*., 2021; Tang *et al*., 2022; Zhang *et al*., 2020a). Surprisingly, lavendustin A not only induces a defence response to efficiently control soil‐borne diseases but also significantly promotes the growth of Arabidopsis and tobacco, which suggests that lavendustin A also targets growth‐related proteins to improve plant growth. FER mutations have been shown to shape a specialized Arabidopsis root microbiota to promote next‐generation plant growth and alleviate Pi starvation (Song *et al*., 2021; Tang *et al*., 2022). The role of lavendustin A in the composition of the plant root microbiome is worth exploring. The effect of lavendustin A on crop yield should also be investigated in the future and further identification of other targets and pathways affected by this inhibitor will be of great value in the development of a common strategy for simultaneously promoting plant resistance and motivating plant growth.

## Materials and methods

### Plant materials and growth conditions

*A. thaliana* wild‐type Col‐0, C24, FER mutants *fer‐4* (complete loss of FER function), *fer‐5* (partial loss of FER function), *srn*, and the label material *pFER::FER‐GFP* were obtained from Duan *et al*. (2010). Col‐0 was used as control for *fer‐4*, *−5* and C24 as control for *srn*, respectively. The MAMP response reporter lines were obtained from Zhou *et al*. (2020). All seeds were surface sterilized and vernalized at 4 °C for 3 days before being grown on 1/2‐strength Murashige and Skoog (MS) media with 0.8% (w/v) sucrose solidified with 1% (w/v) agar for subsequent analysis. Arabidopsis plants were grown under long‐day conditions (16‐h light/8‐h dark cycles) at 22 ± 2 °C with a light intensity of ~170 mmol/m^2^/s. Tobacco K326, rice Nipponbare, and tomato Moneymaker were grown under long‐day conditions at 25 ± 2 °C.

### Protein expression and purification

The FER‐CD (residues 469–895 aa; AT3G51550) and that of the FER homologue NtFER‐CD (residues 467–888 aa; Nitab4.5_0024282) in tobacco were cloned into the pRSF‐Duet vector and then coexpressed in *Escherichia coli* BL21 with *A. thaliana* phosphatase ABA insensitive 2 (*At*ABI2), which was cloned into the pGEX‐4T‐1 vector (Chen *et al*., 2016; Li *et al*., 2018). The FER family proteins HERK1‐CD (residues 427–830 aa; AT3G46290), ANJEA‐CD (residues 428–829 aa; AT5G59700) and THE1‐CD (residues 437–855 aa; AT5G54380; Zhu *et al*., 2021) were cloned into pRSF‐Duet and transformed into BL21. The primers used in this study are listed in Table S3. Protein expression was induced using 0.5 mm isopropyl‐β‐D‐thiogalactopyranoside at 16 °C with shaking at 110 rpm for 18 h. After purification with a Ni^2+^ affinity column (Smart Life Sciences, SA004250), the target protein was exchanged into 50 mm HEPES buffer (pH 7.5). The protein was detected by sodium dodecyl sulphate‐polyacrylamide gel electrophoresis (SDS‐PAGE), and the concentration was determined using a bicinchoninic acid (BCA) assay.

### Measurement of kinase activity

We studied kinase activity based on autophosphorylation ability. The kinase activity of FER‐CD, HERK1‐CD, ANJEA‐CD, THE1‐CD and NtFER‐CD was measured using a Kinase‐Lumi™ Luminescent Kinase Assay Kit (Beyotime, S0150S; Baki *et al*., 2007). After optimizing the protein and ATP concentration and reaction time, the specific steps were determined as follows: 0.5 μm protein was incubated at room temperature for 10 min in 50 μL of assay buffer (50 mm HEPES, 10 mm MgCl_2_, 10 mm MnCl_2_, and 1 mm EGTA, pH 7.5) containing 2.5 μm ATP. After the addition of 50 μL of detection reagent, the mixture was incubated for 10 min. ATP consumption (μm), which represents the kinase activity per 0.5 μm protein, was monitored by chemiluminescence detection using a Multiskan Spectrum spectrophotometer (Thermo Scientific, FLUOROSKAN ASCENT FL). The assay was calibrated against an ATP standard curve.

### High‐throughput screening of kinase inhibitors

In a 96‐well plate, 1494 small molecules from the kinase inhibitor library (Table S4; MedChemExpress, Shanghai, China) were used at a final concentration of 5 μm in the assay buffer for the treatment of 0.5 μm protein for 30 min. After the addition of ATP, the kinase activity was assayed. The inhibitor solvent DMSO (0.05%; v/v) was used as a negative control. A kinase activity inhibition rate of 65% was used as the minimum standard to identify small molecules that could effectively inhibit the activity of FER, with the expectation that appropriate lead compounds would be obtained with this moderately preferred inhibition rate. Three independent experiments were performed to validate FER inhibitors obtained by high‐throughput screening. The effects of FER inhibitors on the kinase activity of HERK1, ANJEA and THE1 were determined, and inhibitors with certain specificity to FER (reversine and lavendustin A) and high efficiency in inhibiting the kinase activity of FER family proteins (cenisertib and staurosporine) were selected as representatives for subsequent experiments.

### *In vivo* FER phosphorylation assay

The anti‐pS701, Y648 and Y704‐FER antibodies were generated by ABclonal (Wuhan, China). Phosphopeptide C‐VVKG‐(p‐S)‐FG (for anti‐pS701), RGLH‐(p‐Y)‐LHTG‐C (for anti‐pY648) and FG‐(p‐Y)‐LDPEYF‐C (for anti‐pY704) were synthesized and used as an antigen to immunize rabbit and generate the polyclonal anti‐phosphorylation antibody. Phosphorylation‐non‐specific antibody were removed using peptide C‐VVKGSFG (for anti‐pS701), RGLHYLHTG‐C (for anti‐pY648) and FGYLDPEYF‐C (for anti‐pY704).

Since the background phosphorylation level of FER in Arabidopsis is difficult to detect, we used RALF1 to activate the phosphorylation of FER. Six‐day‐old *pFER::FER‐GFP* seedlings were treated with liquid 1/2 MS medium containing 5 μm FER inhibitor or control for 12 h, the seedlings were incubated for 20 min in a medium containing 0.2 μm synthetic RALF1. Total protein of the seedlings was extracted and analysed by SDS‐PAGE and immunoblotting with the anti‐pS701, anti‐pY648, anti‐pY704 and anti‐GFP from ABclonal.

### FER homology analysis, modelling and molecular docking

The sequences of FER homologous proteins in tobacco (NtFER), rice (OsFLR1, Os03g21540; OsFLR2, Os01g56330), tomato (SlFER, Solyc09g015830), strawberry (*Fragaria* × *ananassa*; FaFER, KX374343), soybean (GmFER, GLYMA_09G273300) and apple (*Malus domestica*; MdFER, KY435591) were downloaded from the Solanaceae Genomics Network or National Center for Biotechnology Information databases. A multiple sequence alignment of the CDs of these homologues, together with the CD of FER, was conducted using the website http://tcoffee.crg.cat/apps/tcoffee/index.html, and homology modelling was performed using the website https://swissmodel.expasy.org. The FER crystal structure was obtained from Kong *et al*. (2022). The RMSD of the spatial structure of NtFER, OsFLR1, and SlFER compared with that of FER was measured using PyMOL software. According to ‘Tutorials for DOCK 6.9 (http://dock.compbio.ucsf.edu/DOCK_6/tutorials/index.htm)’, Chimera and PyMOL software and R were used for molecular docking analysis of the inhibitors with FER and its homologues. ATP and the inactive small molecule lapatinib were used as positive and negative controls, respectively.

### Microscale thermophoresis (MST) assay and concentration dependence test

For the MST assay, FER‐CD protein (10 μm) was labelled with a fluorescent dye using the MO‐L011 Monolith Protein Labeling Kit RED‐NHS (NanoTemper, Munich, Germany). Labelled FER‐CD (100 nM) was mixed with varying concentrations of FER inhibitor (ranging from 0.0015 to 100 μm) in PBS containing 0.05% Triton X‐100. ATP and lapatinib were used as positive and negative controls, respectively. Approximately, 4–6 μL of each sample was loaded in a fused silica capillary. Measurements were performed at 25°C using a Monolith NT.115 instrument (NanoTemper) at a constant LED power of 20% and the MST power of the medium. The data were then analysed using MO. Affinity Analysis v2.3 NT software to determine the interaction parameters. Signal‐to‐noise ratios above 10 were considered significant, as suggested by NanoTemper. Data point binding curves from three independent MST measurements, which indicate the fraction of inhibitor‐ or ATP‐bound FER‐CD (▵Normal/Amplitude) at different inhibitor or ATP concentrations, are shown and indicate the calculated fits. The error bars represent the SEs of three independent measurements.

For the concentration dependence test, five concentrations (0.5, 1, 2.5, 5 and 10 μm) of FER inhibitors were used to determine the inhibitory effect on FER‐CD kinase activity. The corresponding concentration of DMSO was used as a control.

### Analysis of the roles of FER inhibitors in soil‐borne diseases

After an overnight incubation in B medium (1% peptone, 0.1% tryptone, 0.1% yeast extract and 2.5% glucose), the *R*. *solanacearum* strain CQPS‐1 was collected. To assay the effects of FER inhibitors against bacterial wilt in Arabidopsis seedlings, six‐day‐old Col‐0 seedlings were pretreated for 24 h in liquid 1/2 MS medium that included 5 μm FER inhibitor in a 12‐well plate (Voges *et al*., 2019). DMSO and lapatinib were used as negative controls in this study except where specified otherwise. *R. solanacearum* was then added to the culture solution to obtain a concentration of 3 × 10^7^ colony‐forming units (CFUs)/mL (OD_600_ = 0.01), and the same amount of ddH_2_O was added as a blank control in all inoculation assays in this study. The cotyledon phenotype was observed daily. Each treatment contained 12 seedlings, and three replicates were included.

To assess the effects of FER inhibitors against tobacco and tomato bacterial wilt, 3 mL of 5 μm FER inhibitor was applied to the roots of four‐week‐old seedlings, and the same amount of inhibitor was added 1 day later. One day later, a cut was made in the soil 1 cm from the stem, and the plant roots were inoculated with 10 mL of *R. solanacearum* (3 × 10^8^ CFU/mL). The assay was performed in triplicate at 28 °C using 30 individual tobacco plants or 18 individual tomato plants per treatment. The plant disease phenotype was observed, visual assessments of disease symptoms (determination of a disease index) was performed according to a scale ranging from ‘0’ to ‘4’ as described previously (Vailleau *et al*., 2007), the disease severity index (DSI) was determined, and the amount of bacterial colonization was measured by plate counting (Wu *et al*., 2019). The disease severity was classified into the following five grades: 0, all leaves were healthy; 1, a quarter of the leaves had wilted; 2, approximately half of the leaves had wilted; 3, three‐quarters of the leaves had wilted; and 4, the entire plant had withered.

$$\text{Disease severity index}\;\left(\mathrm{DSI}\right)=\frac{\sum \text{number of disease plants}\times \text{symptom stage}}{\text{Total number of plants}\times \text{highest symptom stage}}\times 100\;$$

For the rice root‐knot nematode disease assay, *M. incognita* egg masses were collected from *Ipomoea aquatica* roots, sterilized with 0.5% NaOCl for 3 min and washed with large volumes of sterile water. The clean eggs were incubated on a sieve and submerged in sterile water at 26 °C for 2 days. Freshly hatched second‐stage juveniles (pre‐J2s) were sterilized with 0.005% HgCl_2_ for 5 min and rinsed with sterile water three times. Two‐week‐old rice was grown in sand with water‐retaining gel and watered twice with 3 mL of 5 μm FER inhibitors as described above. One day later, 800 pre‐J2s were inoculated on the rice roots at 26 °C. The assay was performed in triplicate using 18 individual plants per treatment. The numbers of nematodes at different stages (parasitic second stage, par‐J2; parasitic third stage, par‐J3; parasitic fourth stage, par‐J4), galls and females were determined using acid fuchsin (Sangon Biotech, Shanghai, China) staining (Zhang *et al*., 2020a) and a BX53 microscope (Olympus, Japan).

### RNA‐seq analysis

Four‐week‐old tobacco was irrigated twice with 3 mL of 5 μm lavendustin A and reversine. DMSO was used as a negative control, and four replicates of each treatment were included in the analysis. After 1 day, the tobacco roots were collected, and total RNA was extracted using a mirVana miRNA Isolation Kit (Ambion, AM1561). RNA integrity was assessed using an Agilent 2100 Bioanalyzer (Agilent Technologies, Santa Clara, CA). Libraries were constructed and sequenced using the Illumina sequencing platform (Illumina HiSeq X Ten) by OE Biotech Co., Ltd. (Shanghai, China). The raw data were processed using Trimmomatic v0.36 (Bolger *et al*., 2014). The clean reads were obtained by removing the low‐quality reads and were mapped to the tobacco genome (Nitab_v4.5) using HISAT2 v2.2.1.0 (Kim *et al*., 2015). Bioinformatics analysis was performed according to Wang, Yang *et al*. (2020). Three items, that is ‘immune system process’, ‘antioxidant activity’ and ‘growth’, were extracted from level 2 of the GO functional classification results, and two items, ‘immune system’ and ‘environmental adaptation’, were extracted from level 2 of the KEGG functional classification results (GO database: http://geneontology.org/; KEGG database: http://www.genome.jp/kegg/). The raw RNA‐seq data were uploaded to the NCBI database under accession number PRJNA760981.

### Statistical analysis

Three biological replicates with at least three technical replicates were used in each assay. The mean and standard deviation (SD) values of the replicates were calculated using Microsoft Excel™ software (Microsoft Corp., Redmond, WA). One‐way analysis of variance (ANOVA) (Tukey's HSD test) and Student's *t*‐test were conducted using SPSS software (ver. 23.0; IBM China Company Ltd., Beijing, China) to determine the statistical significance. *P* < 0.05 was considered to indicate a significant difference.

## Conflicts of interests

The authors declare that they have applied for patents on the use of the FER inhibitors reversine, lavendustin A, and staurosporine for controlling plant soil‐borne diseases.

## Author contributions

F.Y., W.P., J.C. and H.‐B.L. conceived the project; H.‐B.L. and F.Y. designed research; H.‐B.L., X.L., J.C., L.‐L.J. and X.Z. performed research; H.‐B.L., D.W., L.W., A.Y. and C.G. analysed data; H.‐B. L. and F.Y. wrote the paper; all authors reviewed and approved the manuscript for publication.

## Supporting information


**Figure S1** Establishment of an *in vitro* high‐throughput screening system for FER kinase inhibitors.
**Figure S2** Chemical structures of 33 FER inhibitors.
**Figure S3** *In vivo* FER phosphorylation assay and multiple sequence alignment of FER and its homologues in multiple crop species and the concentration dependence of four FER inhibitors.
**Figure S4** Effects of FER inhibitors and FER mutations on the resistance of Arabidopsis to *Ralstonia solanacearum*.
**Figure S5** Effect of FER inhibitors on the growth of Arabidopsis seedlings and on the pathogens.
**Figure S6** ROS production and MAPK phosphorylation assays.
**Figure S7** Effects of FER inhibitors on the expression of defence‐related genes in Arabidopsis roots.
**Figure S8** Effects of lavendustin A and reversine on the transcriptome in tobacco roots.
**Figure S9** Top 30 KEGG pathways that were coenriched after lavendustin A and reversine treatments compared with DMSO treatment.
**Figure S10** Effects of FER inhibitors on PEPR1 and CERK1 kinase activity and *PDF1.4* expression in Arabidopsis leaves or roots.
**Figure S11** Effects of FER inhibitor application on established bacterial wilt and root‐knot nematode diseases.

**Appendix S1** Details are supplied in the Supporting Information for the experimental programmes for toxicity testing of FER inhibitors to pathogens, the *R. solanacearum* infection assay, plant growth analysis, detection of ROS in plant roots, MAPK phosphorylation analysis and analysis of the expression of defence‐related genes in roots.

**Table S1** GO functional classification of immune‐, antioxidant‐, and growth‐related genes.

**Table S2** Intersection of KEGG enrichment results (upregulated) between the lavendustin A vs. DMSO and reversine vs. DMSO comparisons.

**Table S3** Specific primers used in this study.

**Table S4** Detailed information for the inhibitor library.

## Data Availability

The data that support the findings of this study are available from the corresponding author upon reasonable request.
