# Supplementary material for: A screening of inhibitors targeting the receptor kinase FERONIA reveals small molecules that enhance plant root immunity
Source: Plant Biotechnol J. 2022 Oct 9;21(1):63–77. doi: 10.1111/pbi.13925 (PMC9829398; doi:10.1111/pbi.13925)
Supplement: Supplementary file 1 — Figure S1 Establishment of an in vitro high‐throughput screening system for FER kinase inhibitors. Figure S2 Chemical structures of 33 FER inhibitors. Figure S3 In vivo FER phosphorylation assay and multiple sequence alignment of FER and its homologues in multiple crop species and the concentration dependence of four FER inhibitors. Figure S4 Effects of FER inhibitors and FER mutations on the resistance of Arabidopsis to Ralstonia solanacearum. Figure S5 Effect of FER inhibitors on the growth of Arabidopsis seedlings and on the pathogens. Figure S6 ROS production and MAPK phosphorylation assays. Figure S7 Effects of FER inhibitors on the expression of defence‐related genes in Arabidopsis roots. Figure S8 Effects of lavendustin A and reversine on the transcriptome in tobacco roots. Figure S9 Top 30 KEGG pathways that were coenriched after lavendustin A and reversine treatments compared with DMSO treatment. Figure S10 Effects of FER inhibitors on PEPR1 and CERK1 kinase activity and PDF1.4 expression in Arabidopsis leaves or roots. Figure S11 Effects of FER inhibitor application on established bacterial wilt and root‐knot nematode diseases. [file PBI-21-63-s005.docx]

**
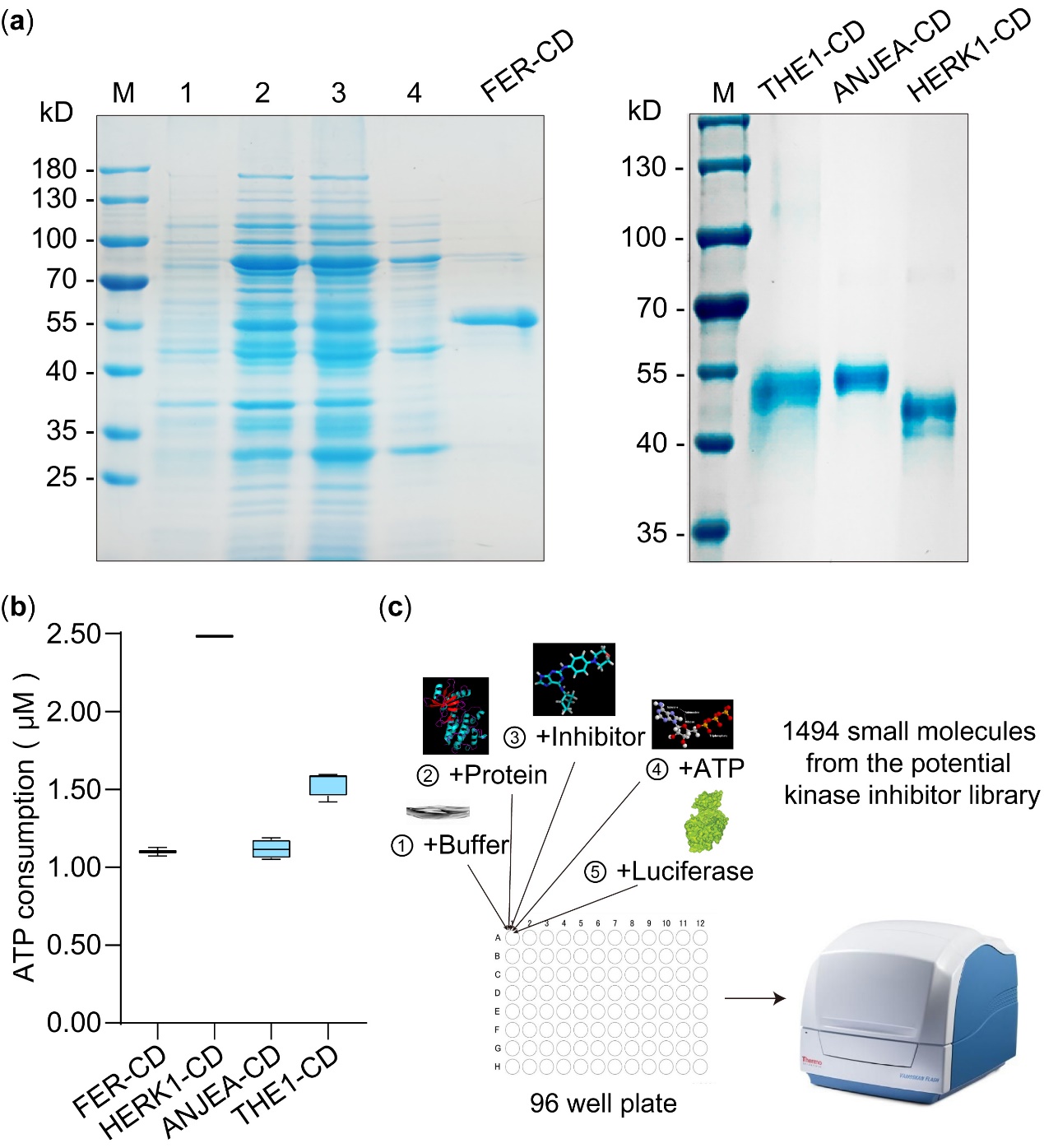
**

**Fig. S1. Establishment of an *in vitro* high-throughput screening system for FER kinase inhibitors. (a)** Prokaryotic expression and purification of the cytoplasmic domain of Arabidopsis receptor kinase FER and its homologs HERK1, ANJEA, and THE1. M: protein marker; 1: bacterial protein before IPTG induction; 2: bacterial protein after IPTG induction; 3: protein in the supernatant after sonication of the induced bacteria; 4: protein in the washing buffer after washing. **(b)** Kinase activity (autophosphorylation) of FER, HERK1, ANJEA, and THE1. The consumption of ATP by 0.5 μM protein in 10 min was used to indicate the level of kinase activity (μM). In boxplots, the middle line represents the median, box edges delimit lower and upper quartiles, and whiskers show the highest and lowest data points (n = 4). The assay was repeated three times with similar results. **(c)** High-throughput screening system for FER inhibitors.

**
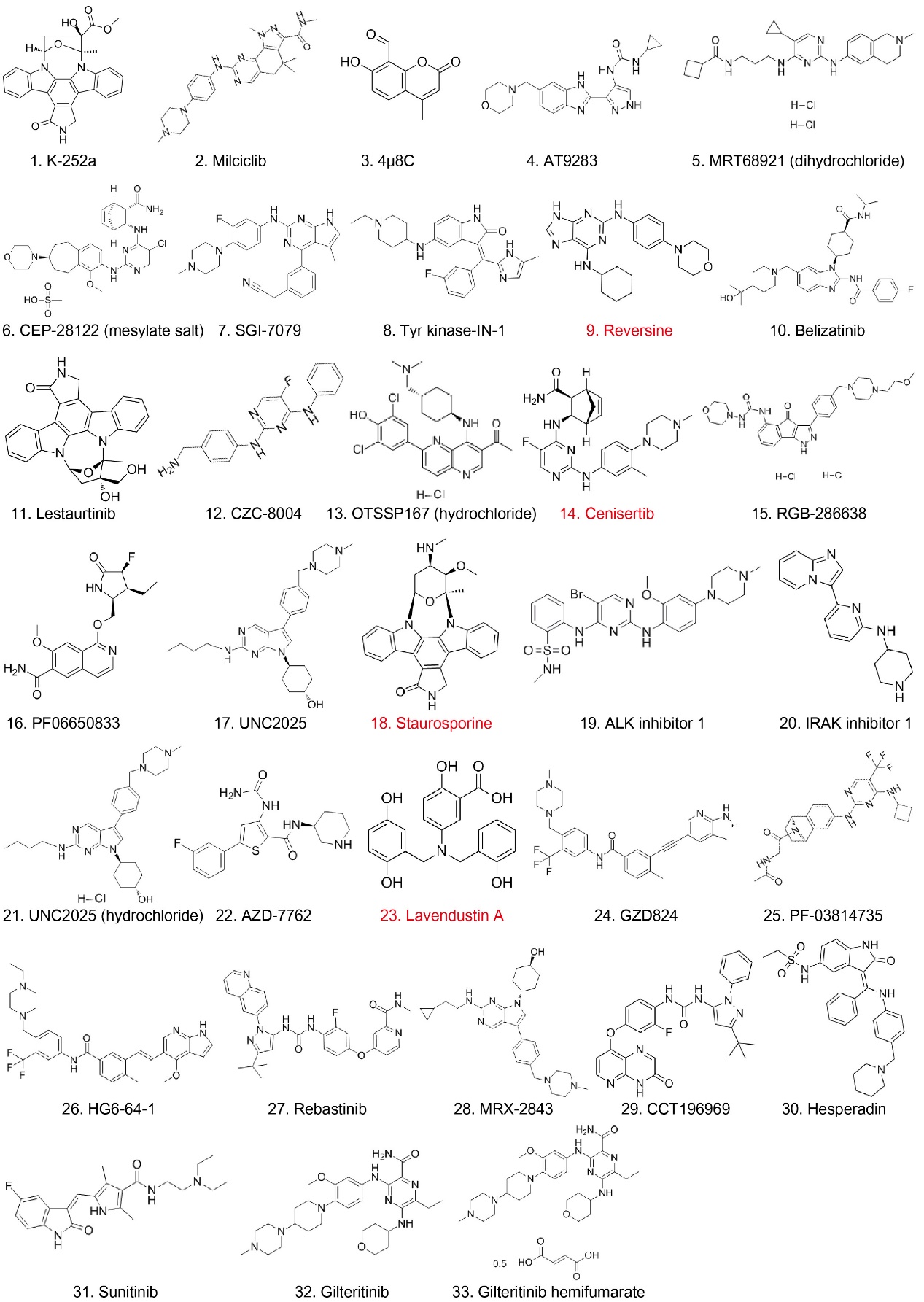
**

**Fig. S2.** **Chemical structures of 33 FER inhibitors.** The FER inhibitors subjected to further investigation are marked in red.

**
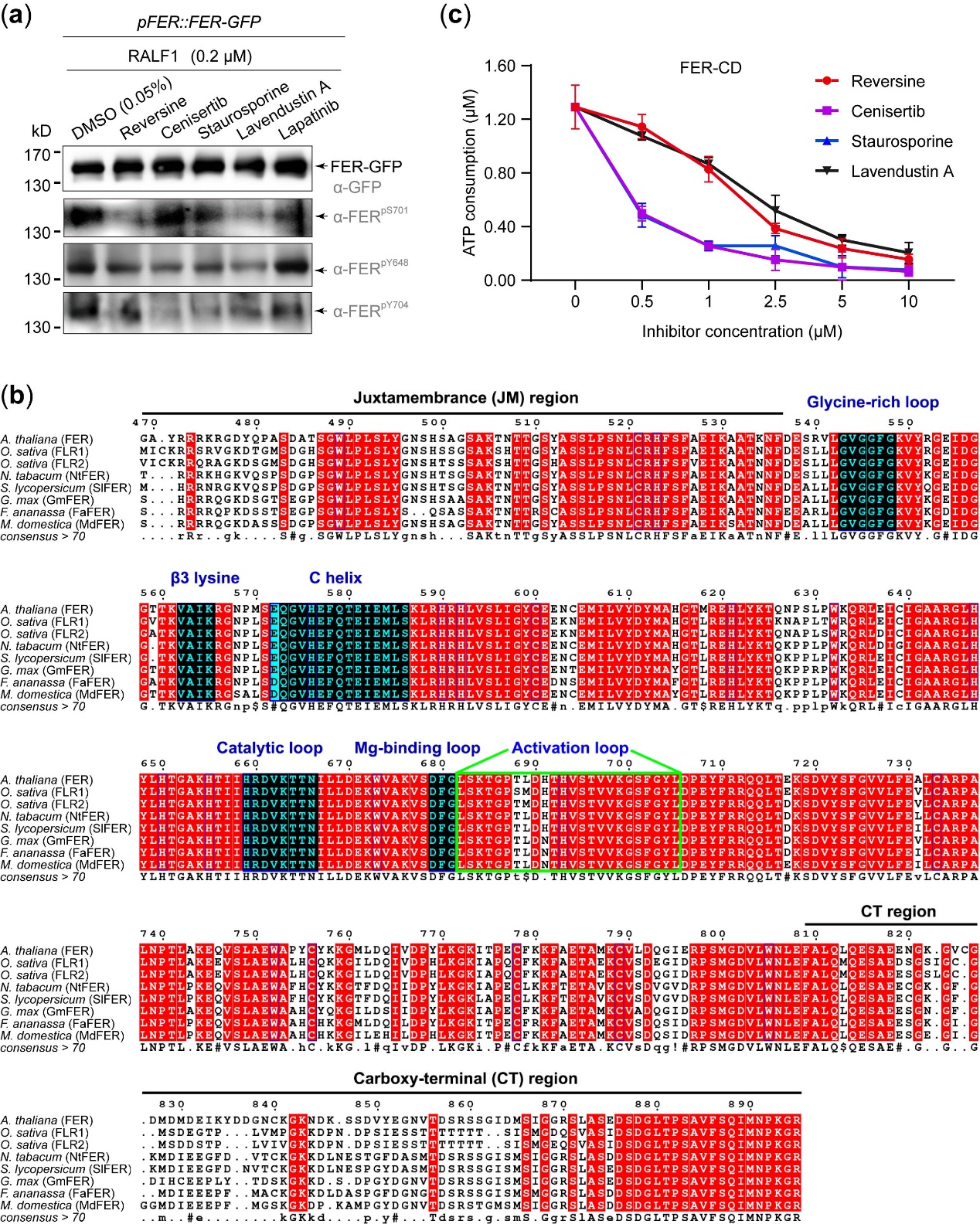
**

**Fig. S3.** ***In vivo* FER phosphorylation assay and multiple sequence alignment of FER and its homologs in multiple crop species and the concentration dependence of four FER inhibitors. (a)** 5 μM of FER inhibitors reduced RALF1-induced FER phosphorylation levels in Arabidopsis. FER-specific anti-phosphorylation antibodies anti-pS701, anti-pY648, and anti-pY704 were used to detect FER phosphorylation levels and anti-GFP was used to detect FER protein expression levels. DMSO (0.05%) and inactive small molecule lapatinib were used as negative controls. **(b)** The cytoplasmic domains of FER and its homologs in rice (*Oryza sativa* L.), tobacco (*Nicotiana tabacum* L.), tomato (*Lycopersicon esculentum* Miller.), soybean (*Glycine max* (Linn.) Merr.), strawberry (*Fragaria* × *ananassa* Duch.), and apple (*Malus domestica*) were tested. The key components in the kinase structures, including the glycine-rich loop, β3 lysine, C helix, catalytic loop, Mg-binding loop, and activation loop, are shown in dark blue. **(c)** The inhibitory effect of different concentrations of the FER inhibitors reversine, cenisertib, staurosporine, and lavendustin A on FER-CD kinase activity (ATP consumption, μM). The data are presented as the means ± SDs (n = 4). The assay was repeated three times with similar results.


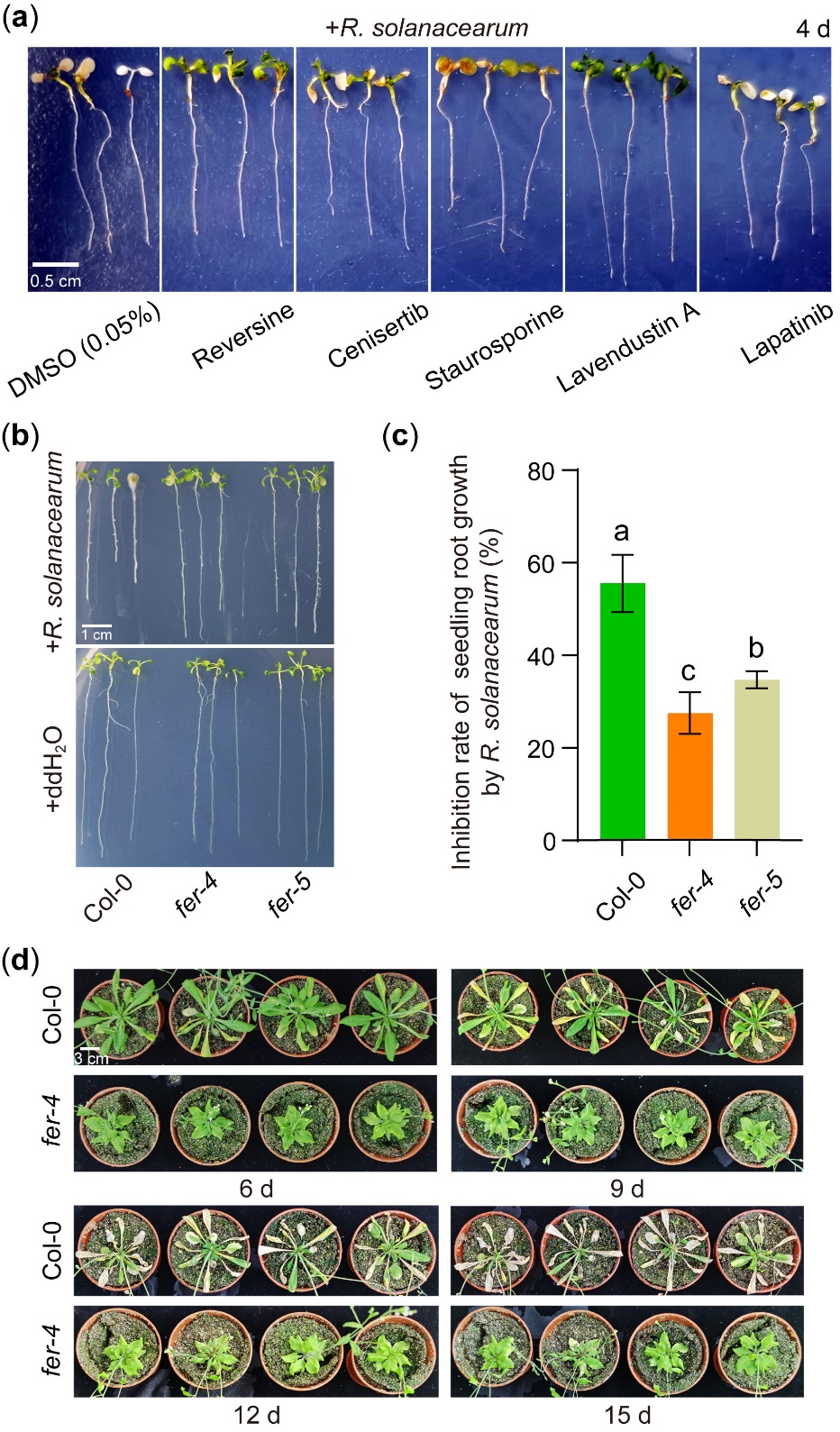


**Fig. S4. Effects of FER inhibitors and FER mutations on the resistance of Arabidopsis to *Ralstonia solanacearum*.** **(a)** Representative images of Arabidopsis seedlings pretreated with 5 μM FER inhibitors 4 days after inoculation with *R. solanacearum*. DMSO (0.05%) and lapatinib were used as negative controls. The assay was repeated three times with consistent results. **(b and d)** Representative images of seedlings **(b)** and four-week-old plants **(d)** of different Arabidopsis genotypes infected with *R. solanacearum*. **(c)** Rate of the inhibition of the root length of Col-0, *fer-4*, and *fer-5* seedlings infected with *R*. *solanacearum* for 3 days. The data are presented as the means ± SDs of three biological replicates. Different letters above the bars indicate significant differences (*p* < 0.05) determined by ANOVA with Tukey’s HSD test.

**
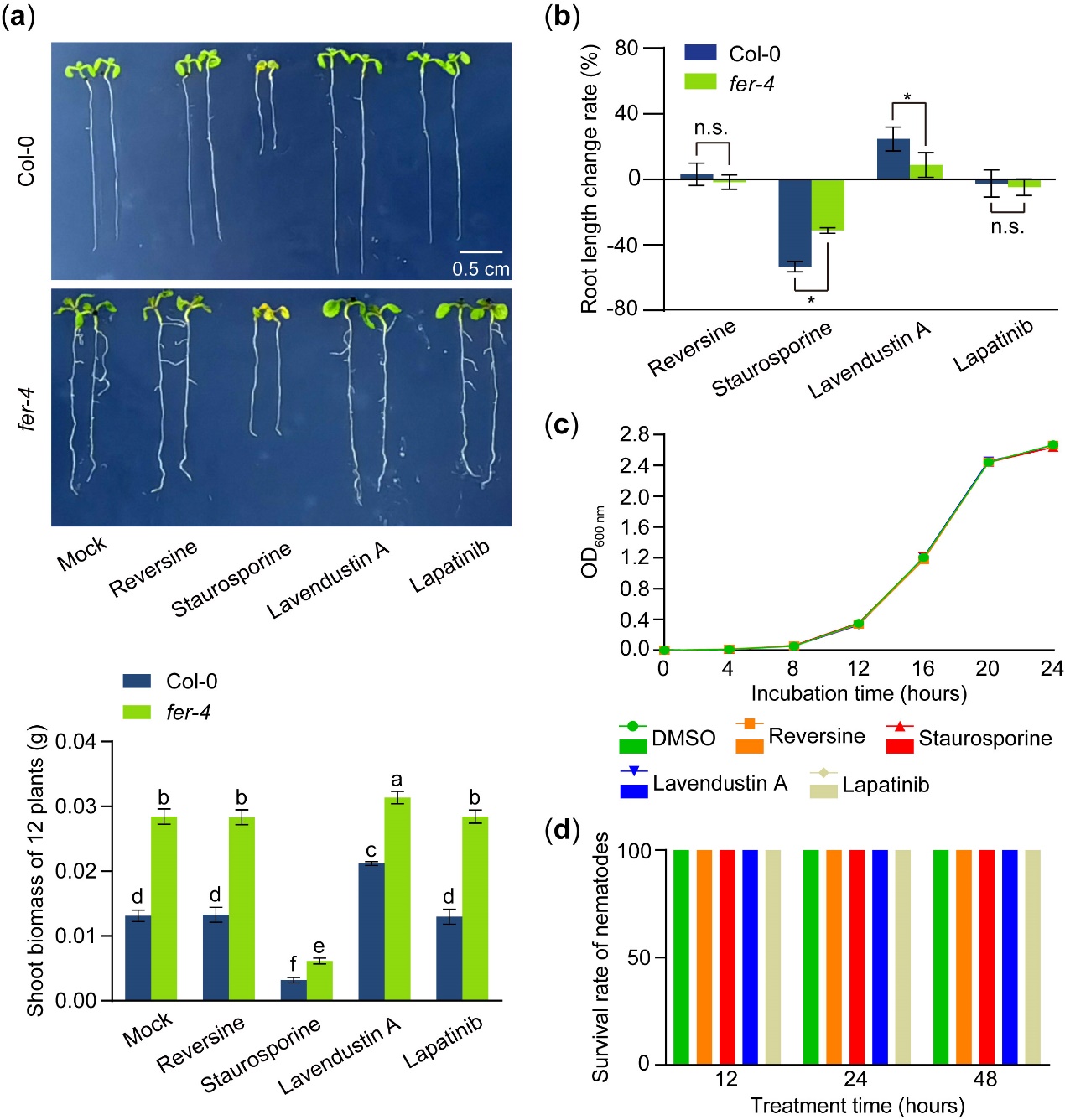
**

**Fig. S5. Effect of FER inhibitors on the growth of Arabidopsis seedlings and on the pathogens.** **(a)** Representative photographs and shoot biomass of Col-0 and *fer-4* seedlings treated with 5 μM FER inhibitors for 3 days. **(b)** Rate of the root length change caused by FER inhibitors compared with the control DMSO. **(c and d)** FER inhibitors do not affect the proliferation of *R. solanacearum* **(c)** or the survival of *Meloidogyne incognita* **(d)**. The data are presented as the means ± SDs of three biological replicates. In **(a)**, different letters above the bars indicate significant differences (*p* < 0.05) determined by ANOVA with Tukey’s HSD test. In **(b)**, **p* < 0.05; n.s., not significant (Student’s *t*-test).

**
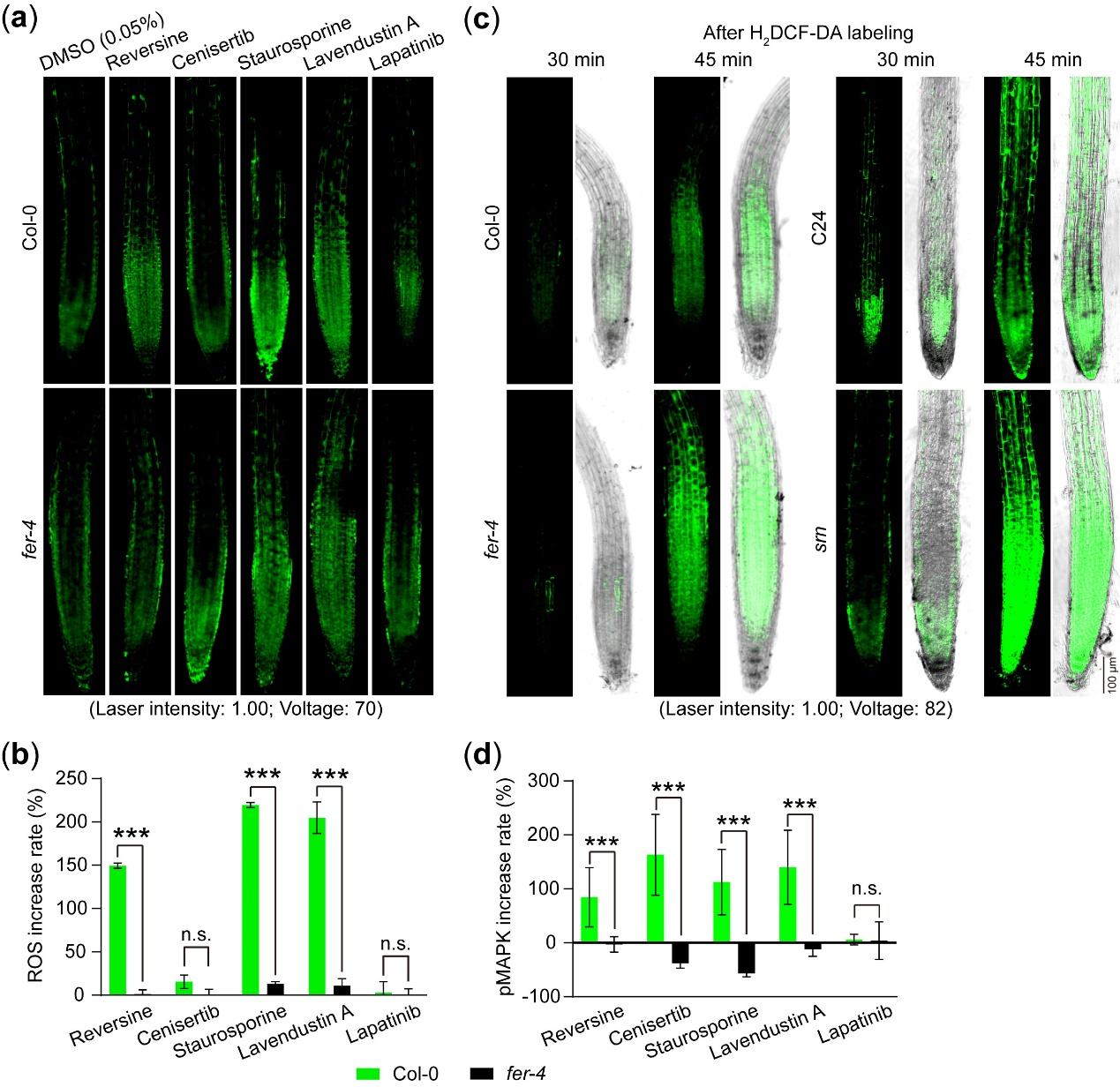
**

**Fig. S6. ROS production and MAPK phosphorylation assays.** **(a)** Single-channel fluorescence images of the representative images shown in Figure 6A. **(b and d)** Rates of the increases in ROS fluorescence intensity **(b)** and MAPK phosphorylation level **(d)** in Col-0 and *fer-4* after treatment with FER inhibitors relative to that after treatment with DMSO. Lapatinib was used as a negative control. The data are presented as the means ± SDs of three biological replicates; ***, *p* < 0.001; n.s., not significant (Student’s *t*-test). **(c)** Representative images of Col-0, *fer-4*, C24, and *srn* roots in inhibitor-free medium at 30 min and 45 min after H_2_DCF-DA labeling. Since H_2_DCF-DA requires sufficient time to be fully deacetylated by cellular esterases to a non-fluorescent compound to react with ROS, after H_2_DCF-DA labeling, we took a time of 45 min as in inhibitor treatment and a shorter time of 30 min for incubation to determine ROS changes in the roots of FER mutants. *fer-4* and *srn* showed weaker ROS levels at 30 min and stronger ROS levels at 45 min than Col-0 and C24.

**
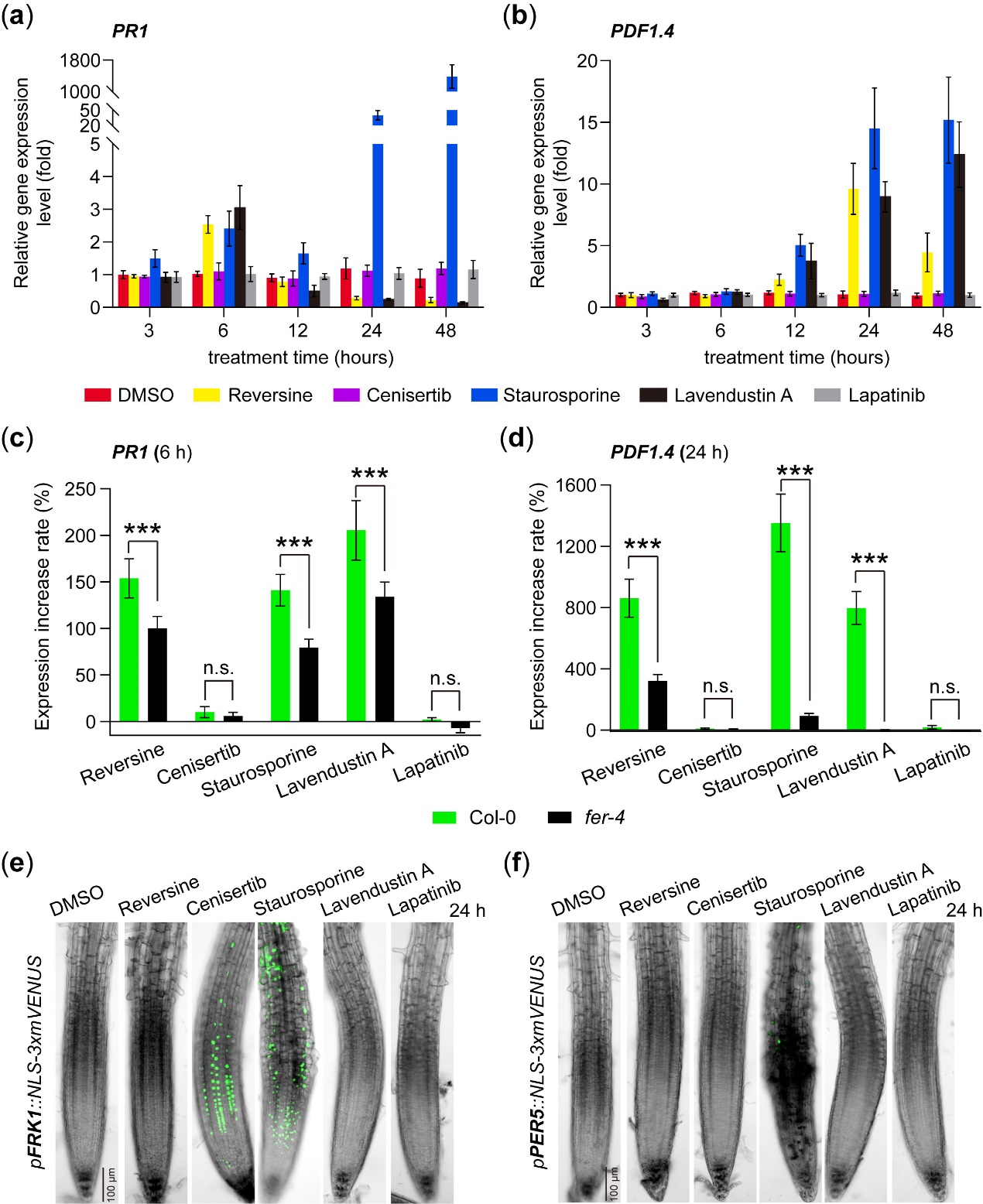
**

**Fig. S7. Effects of FER inhibitors on the expression of defense-related genes in Arabidopsis roots. (a and b)** Relative expression levels of *PR1* **(a)** and *PDF1.4* **(b)** in Arabidopsis Col-0 seedling roots treated with 5 μM FER inhibitors for 3, 6 ,12, 24, or 48 h. DMSO (0.05%) and the inactive small-molecule lapatinib were used as negative controls. **(c and d)** Increased expression rates of *PR1* **(c)** and *PDF1.4* **(d)** in Col-0 and *fer-4* seedling roots treated with 5 μM FER inhibitors for 6 or 24 h relative to that after treatment with DMSO. The data are presented as the means ± SDs of three biological replicates; ****p* < 0.001; n.s., not significant (Student’s *t*-test). **(e** **and f)** Expression pattern of the *FRK1* marker **(e)** and *PER5* marker **(f)** in the root tip of Col-0 treated with 5 μM FER inhibitors for 24 h. Representative images are presented.

**
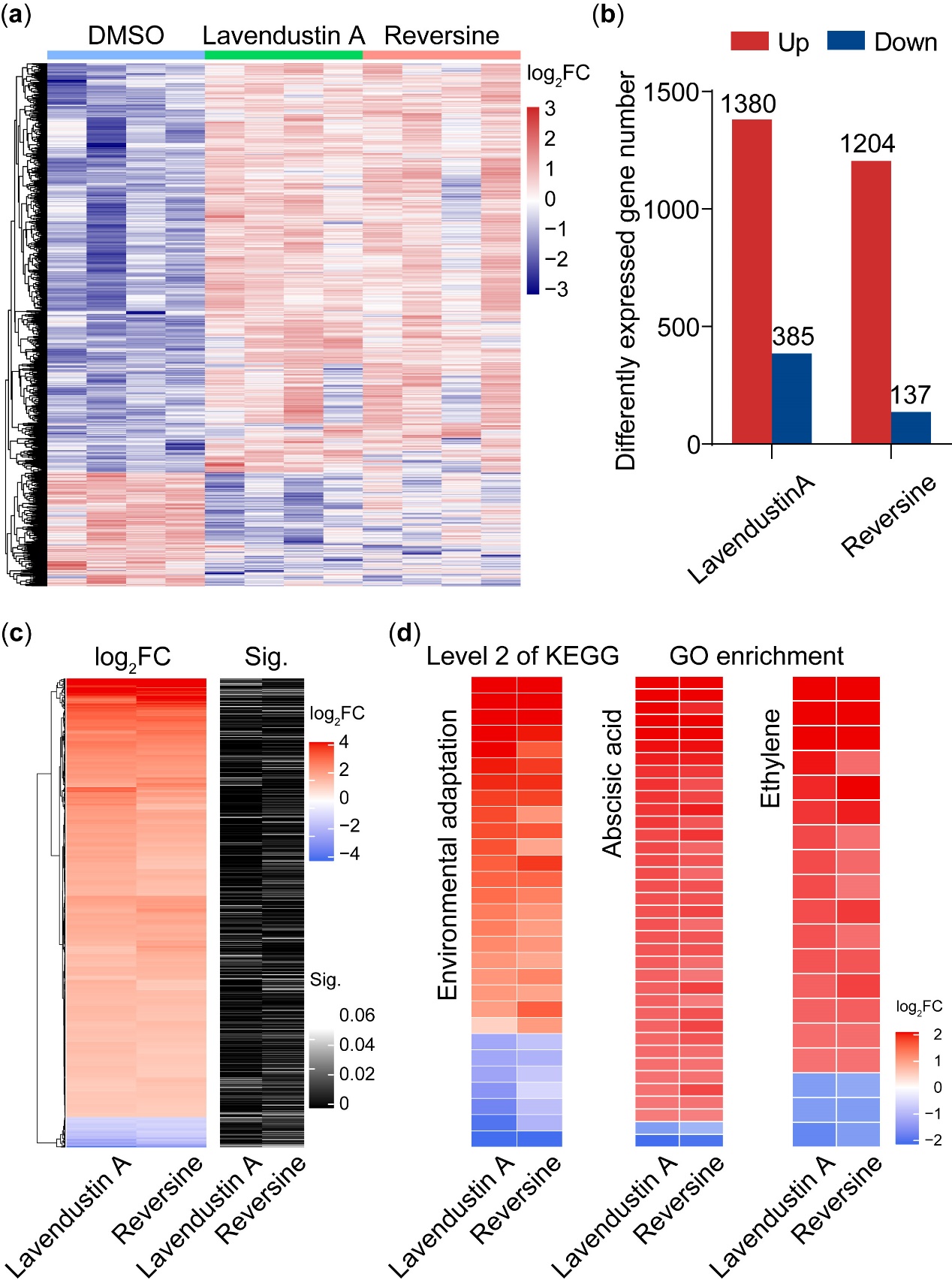
**

**Fig. S8. Effects of lavendustin A and reversine on the transcriptome in tobacco roots. (a)** Heatmap of differentially expressed genes (DEGs) shared by the lavendustin A and reversine treatment groups compared with the control DMSO treatment group (n = 4). **(b)** Summary of significant DEGs showing a fold change > 2 (upregulated) or < 0.5 (downregulated) (*p* < 0.05) after the lavendustin A and reversine treatments compared with the control DMSO. **(c)** Heatmap of fold changes in the expression of DEGs after lavendustin A treatment or reversine treatment compared with that obtained with DMSO treatment. Sig. indicates *p* < 0.05 for each treatment. **(d)** Fold change of DEGs related to environmental adaptation from level 2 of the KEGG functional classification results and defense hormones enriched by GO.


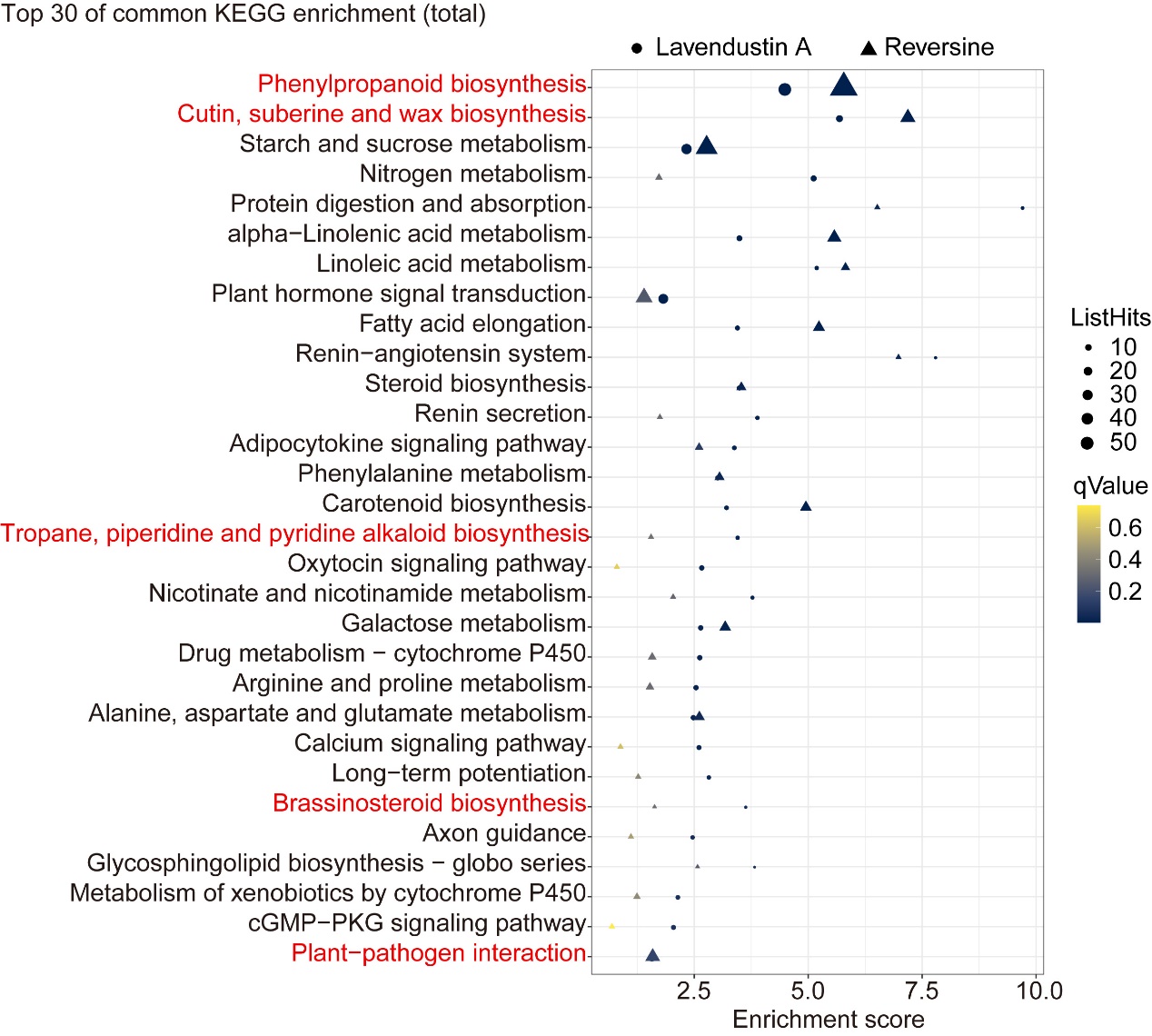


**Fig. S9. Top 30 KEGG pathways that were coenriched after lavendustin A and reversine treatments compared with DMSO treatment.**

**
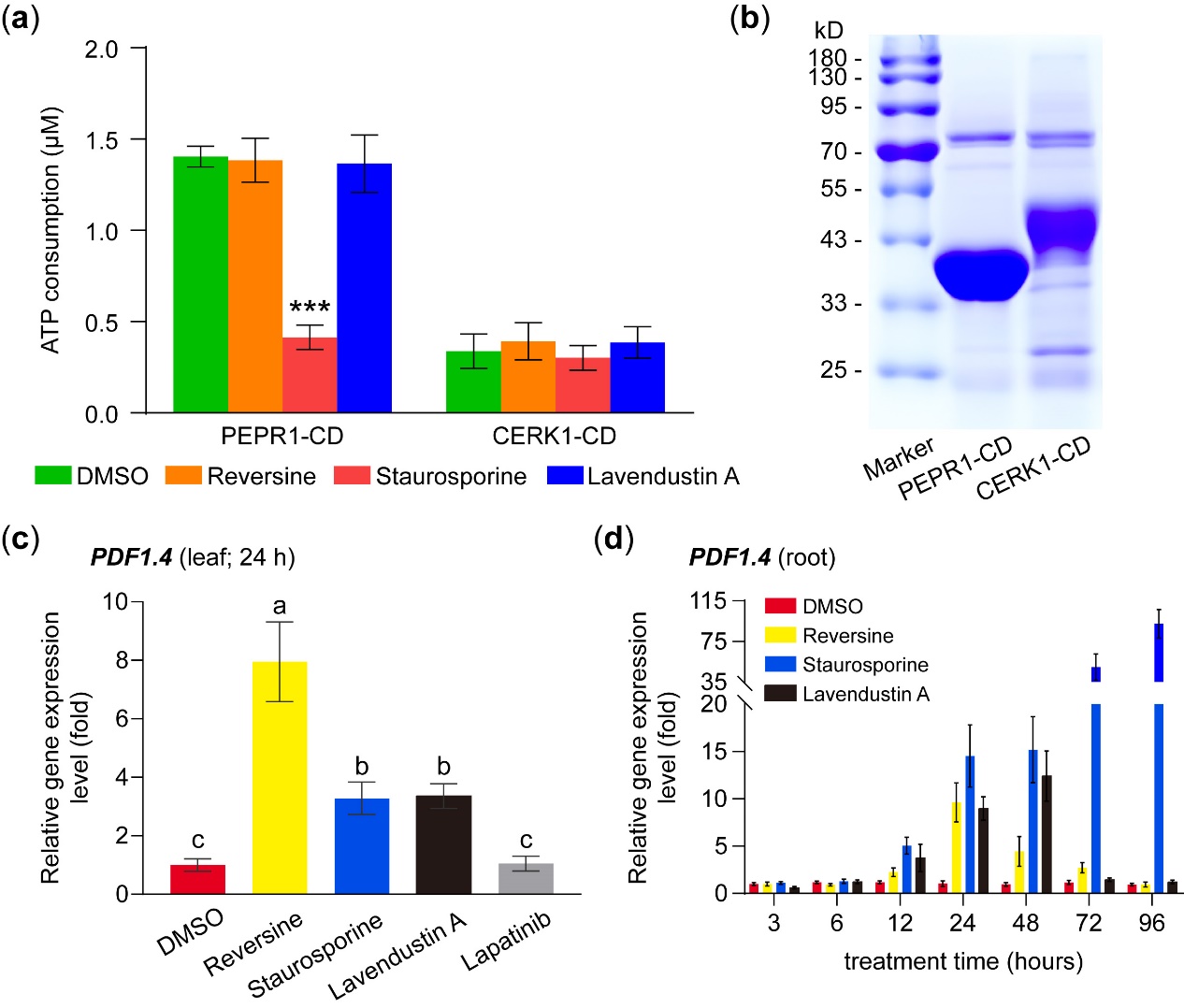
**

**Fig. S10. Effects of FER inhibitors on PEPR1 and CERK1 kinase activity and *PDF1.4* expression in Arabidopsis leaves or roots. (a)** Effects of FER inhibitors on the kinase activity of the Arabidopsis receptor kinase PEPR1 and CERK1 cytoplasmic domains (CDs). PEPR1-CD: residues 791–1123 aa; AT1G73080. CERK1-CD: residues 254–617 aa; AT3G21630. DMSO (0.05%; v/v) was used as a negative control. The data are shown as the means ± SDs (n = 4). The assay was repeated three times with similar results. ***, *p* < 0.001 (Student’s *t*-test). **(b)** Protein purification of PEPR1-CD and CERK1-CD. **(c)** Relative expression levels of *PDF1.4* in Arabidopsis Col-0 rosette leaves treated with 5 μM FER inhibitors for 24 h. DMSO and inactive lapatinib were used as negative controls. **(d)** Relative expression levels of *PDF1.4* in Col-0 seedling roots treated with 5 μM FER inhibitors for 3, 6 ,12, 24, 48, 72, or 96 h. In **(c and d)**, the data are presented as the means ± SDs of three biological replicates. In **(c)**, different letters above the bars indicate significant differences (*p* < 0.05) determined by ANOVA with Tukey’s HSD test.

**
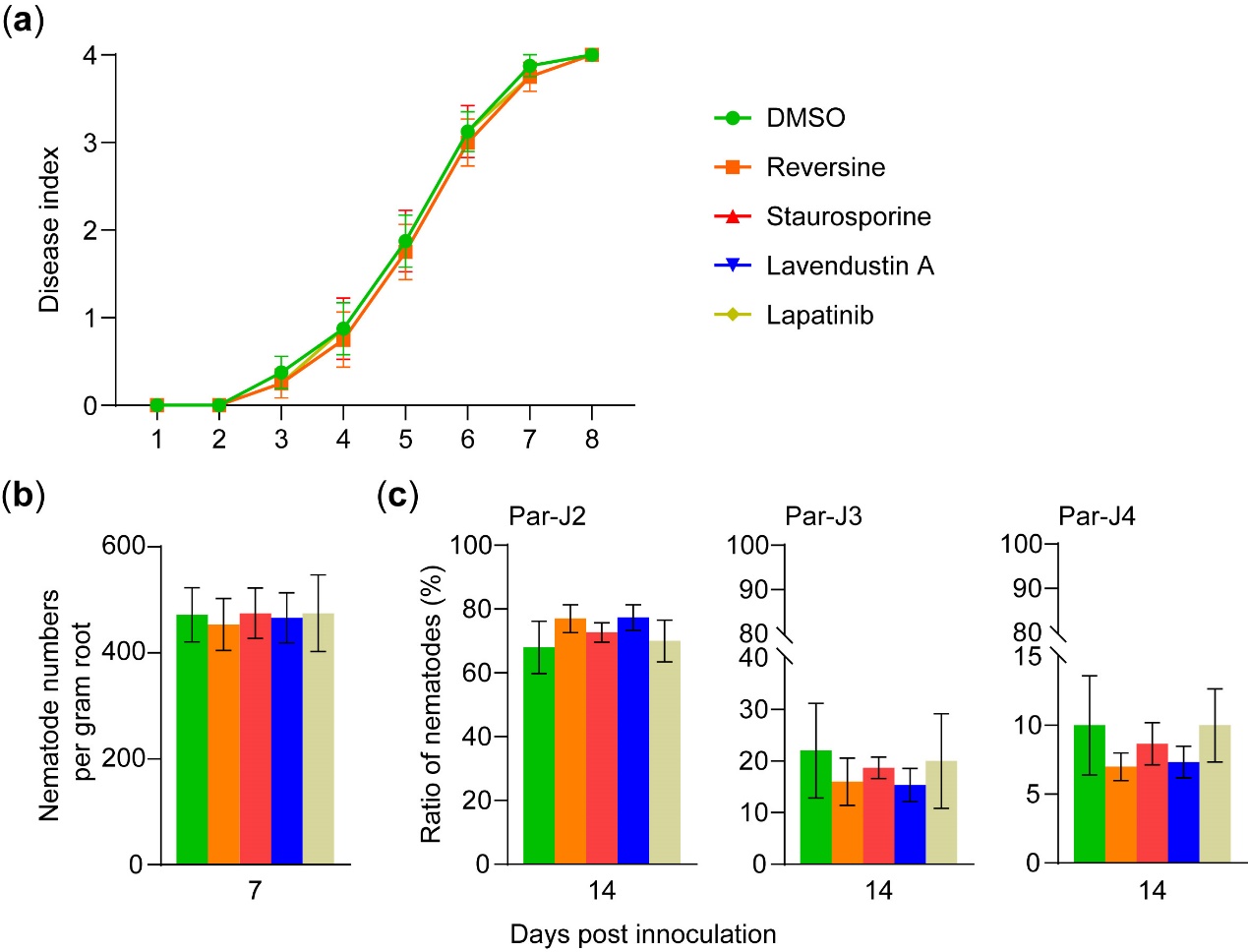
**

**Fig. S11. Effects of FER inhibitor application on established bacterial wilt and root-knot nematode diseases. (a)** Disease index of tobacco on the indicated days after inoculation with *R. solanacearum*. Four-week-old tobacco roots were irrigated twice with 5 μM FER inhibitors two days after inoculation with the bacteria. **(b)** Number of nematodes per gram of root at 7 days after inoculation. Two-week-old rice roots were irrigated with FER inhibitors three days after inoculation with *Meloidogyne incognita*. **(c)** The ratio of par-J2, par-J3, and par-J4 nematodes to the total number of parasitic nematodes at 14 days after inoculation. The assays were performed in triplicate using 18 individual tobacco or rice plants per treatment. DMSO (0.05%; v/v) and lapatinib were used as negative controls. The data shown indicate the means ± SDs of three biological replicates.
