# Supplementary material for: A screening of inhibitors targeting the receptor kinase FERONIA reveals small molecules that enhance plant root immunity
Source: Plant Biotechnol J. 2022 Oct 9;21(1):63–77. doi: 10.1111/pbi.13925 (PMC9829398; doi:10.1111/pbi.13925)
Supplement: Supplementary file 2 — Appendix S1 Details are supplied in the Supporting Information for the experimental programmes for toxicity testing of FER inhibitors to pathogens, the R. solanacearum infection assay, plant growth analysis, detection of ROS in plant roots, MAPK phosphorylation analysis and analysis of the expression of defence‐related genes in roots. [file PBI-21-63-s001.docx]

**Supporting information for materials and methods**

**Toxicity test of FER inhibitors to pathogens**

A growth curve assay was performed for *R. solanacearum*. An overnight culture of 3 × 10^9^ CFU/mL was diluted 1:1000 into 10 mL of B medium containing 5 μM FER inhibitor and then incubated at 28°C with shaking at 180 rpm for 24 h. The optical density was read spectrophotometrically at 600 nm with time interval of 4 h. A survival test was conducted for pre-J2s of *M. incognita*. Approximately 50 nematodes were immersed in ddH_2_O that included 5 μM FER inhibitor in a 12-well plate wrapped with aluminum foil at 26°C for 12, 24, and 48 h. DMSO and lapatinib were used as negative controls. The nematodes were observed under a microscope and were scored as either alive or dead. Dead nematodes were certified by disturbing them with a needle and those that remained immotile and had a straight shape were considered as dead.

***R. solanacearum* infection assay**

Arabidopsis seedlings infected with *R*. *solanacearum* show an inhibited root growth phenotype (Lu *et al*., 2018). For the seedling inoculation assay, six-day-old Col-0, *fer-4*, and *fer-5* seedlings with uniform growth were selected. Five microliters of *R. solanacearum* suspension (3 × 10^7^ CFU/mL) was applied to the seedlings through the deposition of small droplets 1 cm from the root tip. The plants were then grown vertically at 28°C, which is suitable for bacterial but not for Arabidopsis growth, for three days; subsequently, photographs were obtained, and the root length was measured using ImageJ software. Each treatment contained 12 seedlings, and three replicates were performed.

For the four-week-old Arabidopsis, a cut was made in the soil 1 cm from the stem, and 20 mL of *R. solanacearum* (3 × 10^8^ CFU/mL) was inoculated into the soil around the roots of Col-0 and *fer-4*. The infection assay was performed in triplicate using 24 individual Arabidopsis plants of each genotype at 28°C. Every three days, the plant disease was observed, the disease index and DSI were determined, and the amount of bacterial colonization was measured.

**Plant growth analysis**

To assess the growth of Arabidopsis seedlings, Col-0 and *fer-4* were vertically cultured on solid 1/2 MS medium for 6 days, and seedlings with uniform growth were transferred to liquid 1/2 MS medium containing 5 μM FER inhibitor and cultured for 3 days. ddH_2_O (mock) and lapatinib were used as negative controls in growth assays. The treated seedlings were photographed and the root length and the fresh weights of shoots were measured. Each treatment contained 12 seedlings, and three replicates were performed.

To assess the growth of tobacco and rice, one-week-old tobacco and rice with uniform growth were irrigated with 3 mL of 5 μM FER inhibitor. The same treatment was performed 1 day later, and the tobacco and rice were irrigated with water and nutrient solution, respectively, every other day. After 25 days of normal culture, the fresh weights of the shoots and roots of tobacco and rice were measured. The assay was performed in triplicate using 18 individual tobacco or rice plants per treatment.

**Detection of reactive oxygen species (ROS) in plant roots**

Six-day-old Col-0 and *fer-4* seedlings were treated with liquid 1/2 MS medium containing 10 μM ROS fluorescent probe H_2_DCF-DA for 15 min, and the seedlings were subjected to three 5-min washes with the culture solution (Song *et al*. 2021). The seedlings were then incubated for 15 min in a medium containing 5 μM FER inhibitor and subjected to three 5-min washes. Incubation in inhibitor-free medium for the same time (45 minutes after H_2_DCF-DA labeling) or a shorter time (30 minutes after H_2_DCF-DA labeling) was also set for Col-0, *fer-4*, C24, and *srn* seedlings. Each treatment contained 10 seedlings, and three replicates were performed. The ROS fluorescence in the same location in the root tip of the seedlings was imaged using a laser-scanning confocal microscope with a 10× water immersion objective, an excitation wavelength of 488 nm (Nikon A1), and the same laser detection intensity. The integral fluorescence intensity was quantified using ImageJ software after the background was subtracted and the area was unified.

**Mitogen-activated protein kinase (MAPK) phosphorylation analysis**

Six-day-old Col-0 and *fer-4* seedlings were treated with liquid 1/2 MS medium containing 5 μM FER inhibitor for 15 min. Total protein of the seedling roots was extracted and analyzed by SDS-PAGE and immunoblotting with phospho-p44/42 MAPK (Erk1/2) (Thr202/Tyr204) (D13.14.4E) XP rabbit mAb and β-actin (26F7) mouse mAb (Cell Signaling Technology, Boston, MA, USA). The gray values were measured using ImageJ software after subtracting the background. The statistical significance was calculated based on three biological repeats.

**Analysis of the expression of defense-related genes in roots**

Six-day-old Col-0 and *fer-4* seedlings or four-week-old rosette leaves were treated with liquid 1/2 MS medium containing 5 μM FER inhibitor for 3, 6, 12, 24, 48, 72, or 96 h. Total RNA of the seedling roots or the rosette leaves was extracted using TRIzol reagent (TaKaRa, Japan). qRT-PCR was performed using the CFX96 Touch Real-Time PCR Detection System (Bio-Rad, USA) with SYBR Premix ExTaq II (Takara, Japan). *ACTIN2* was used as a reference and relative expression levels (fold changes) were determined with the 2^−ΔΔCt^ method. The primers used for *ACTIN2*, *PR1* (*PATHOGENESIS-RELATED GENE 1*) and *PDF1.4* (*PLANT DEFENSIN 1.4*) are listed in **Table S1.** For the expression analysis of MAMP-induced *FRK1* (*FLG22-INDUCED RECEPTOR-LIKE KINASE 1*) and *PER5* (*PEROXIDASE 5*), the six-day-old reporter lines *pFRK1::NLS-3×mVENUS* and *pPER5::NLS-3×mVENUS* were incubated in liquid 1/2 MS medium containing 5 μM FER inhibitor for 24 h. Each treatment contained 10 seedlings, and three replicates were performed. The green fluorescence in the same location in the seedling roots was imaged using a laser-scanning confocal microscope with 10× water immersion objectives and an excitation wavelength of 488 nm (Nikon A1).

**REFERENCE:**

Lu, H.B., Lema, A.S., Planas-Marques, M., Alonso-Diaz, A., Valls, M. and Coll, N.S. (2018) Type III secretiondependent and -independent phenotypes caused by Ralstonia solanacearum in Arabidopsis roots. Mol. Plant Microbe Interact. 31 , 175–184.
