# Supplementary material for: A screening of inhibitors targeting the receptor kinase FERONIA reveals small molecules that enhance plant root immunity
Source: Plant Biotechnol J. 2022 Oct 9;21(1):63–77. doi: 10.1111/pbi.13925 (PMC9829398; doi:10.1111/pbi.13925)
Supplement: Supplementary file 5 — Table S3 Specific primers used in this study. [file PBI-21-63-s006.doc]

**Table S3.** Specific primers used in this study

| **Primers** | **Sequence (5’ to 3’)** |
| --- | --- |
| FER-CD-F | CCACAGCCAGGATCCGGGTGCTTACCGCAGACGTA |
| FER-CD-R | TTTACCAGACTCGAGCTAACGTCCCTTTGGATTCATGAT |
| HERK1-CD-F | CCACAGCCAGGATCCGTTGTATAAGAAGCGGAAACGTGGCC |
| HERK1-CD-R | TTTACCAGACTCGAGTTATCTTCCTTCAGATTTCACCAGTTGTGAG |
| ANJEA-CD-F | CCACAGCCAGGATCCGTACAAGAAACGAGGACGAGACCAAG |
| ANJEA-CD-R | TTTACCAGACTCGAGTTAACGTCCCTCAGATTTGATCAGCTG |
| THE1-CD-F | CCACAGCCAGGATCCGTATTGCTGTTTGGTTGCTTCAAGGAAG |
| THE1-CD-R | TTTACCAGACTCGAGCTACCTTCCACGAGGATGAACAAG |
| NtFER-CD-F | CCACAGCCAGGATCCGCGCCGCCGGAAACATG |
| NtFER-CD-R | TTTACCAGACTCGAGTTAGCGTCCTTTTGGATTCATG |
| PR1-qF | TGGTCACTACACTCAAGTTGTT |
| PR1-qR | GCTTCTCGTTCACATAATTCCC |
| PDF1.4-qF | ATAGCTTCCACTGAGATGATGG |
| PDF1.4-qR | GAAGTAGCAGAAACATGCGAAA |
| ACTIN2-qF | GGTAACATTGTGCTCAGTGGTGG |
| ACTIN2-qR | AACGACCTTAATCTTCATGCTGC |
